# Bangladesh Environmental Enteric Dysfunction (BEED) study: protocol for a community-based intervention study to validate non-invasive biomarkers of environmental enteric dysfunction

**DOI:** 10.1136/bmjopen-2017-017768

**Published:** 2017-08-11

**Authors:** Mustafa Mahfuz, Subhasish Das, Ramendra Nath Mazumder, M Masudur Rahman, Rashidul Haque, Md Muzibur Rahman Bhuiyan, Hasina Akhter, Md. Shafiqul Alam Sarker, Dinesh Mondal, Syed Shafi Ahmed Muaz, A S M Bazlul Karim, Stephen M Borowitz, Christopher A Moskaluk, Michael J Barratt, William A Petri, Jeffrey I Gordon, Tahmeed Ahmed

**Affiliations:** 1 Nutrition and Clinical Services Division (NCSD), International Centre for Diarrhoeal Disease Research, Bangladesh (icddr,b), Dhaka, Bangladesh; 2 Department of Gastroenterology, Dhaka Medical College and Hospital, Dhaka, Bangladesh; 3 Apollo Hospital, Dhaka, Bangladesh; 4 Department of Paediatric Gastroenterology, Hepatology & Nutrition, Dhaka Shishu Hospital, Dhaka, Bangladesh; 5 Department of Paediatric Gastroenterology & Nutrition, Bangabandhu Sheikh Mujib Medical University (BSMMU), Dhaka, Bangladesh; 6 Division of Infectious Diseases and International Health, University of Virginia, Charlottesville, Virginia, USA; 7 Center for Genome Sciences and Systems Biology, Washington University, St Louis, Missouri, USA; 8 Center for Gut Microbiome and Nutrition Research, Washington University, St. Louis, Missouri, USA

**Keywords:** Stunting, EED, Nutritional intervention, Upper gastro-intestinal endoscopy, Biomarker; Gut microbiota

## Abstract

**Introduction:**

Environmental enteric dysfunction (EED) is a subacute inflammatory condition of the small intestinal mucosa with unclear aetiology that may account for more than 40% of all cases of stunting. Currently, there are no universally accepted protocols for the diagnosis, treatment and ultimately prevention of EED. The Bangladesh Environmental Enteric Dysfunction (BEED) study is designed to validate non-invasive biomarkers of EED with small intestinal biopsy, better understand disease pathogenesis and identify potential therapeutic targets for interventions designed to control EED and stunting.

**Methods and analysis:**

The BEED study is a community-based intervention where participants are recruited from three cohorts: stunted children aged 12–18 months (length for age Z-score (LAZ) <−2), at risk of stunting children aged 12–18 months (LAZ <−1 to −2) and malnourished adults aged 18–45 years (body mass index <18.5 kg/m^2^). After screening, participants eligible for study provide faecal, urine and plasma specimens to quantify the levels of candidate EED biomarkers before and after receiving a nutritional intervention. Participants who fail to respond to nutritional therapy are considered as the candidates for upper gastrointestinal endoscopy with biopsy. Histopathological scoring for EED will be performed on biopsies obtained from several locations within the proximal small intestine. Candidate EED biomarkers will be correlated with nutritional status, the results of histochemical and immunohistochemical analyses of epithelial and lamina propria cell populations, plus assessments of microbial community structure.

**Ethics and dissemination:**

Ethics approval was obtained in all participating institutes. Results of this study will be submitted for publication in peer-reviewed journals.

**Trial registration number:**

ClinicalTrials.gov ID: NCT02812615. Registered on 21 June 2016.

**Strengths and limitations of this study**

- This study will compare a wide array of non-invasive biomarkers of environmental enteric dysfunction (EED) to the diagnostic gold standard: intestinal histopathology.
- A histological scoring system will be developed for identifying EED and describing its severity.
- This study will be one of the largest community-based nutritional interventional studies in improving the growth parameters in children with stunting.
- Due to ethical reasons, collecting small intestinal tissue from healthy population of the same community will not be possible.
- ‘Failure to thrive’ criterion was selected based on assumptions as no data were available.

## Introduction

Environmental enteric dysfunction (EED), previously known as tropical enteropathy and environmental enteropathy, is a subacute inflammatory condition of the small intestinal mucosa with unclear aetiology. EED has been associated with a variety of environmental exposures and host factors and is implicated in growth faltering.^1^ Linear growth faltering typically occurs within the first 2 years of life and after this period, it is difficult to reverse.^2^ Globally, there are more than 160 million stunted children (length for age Z-score (LAZ) <−2).^3^ Previous work in Gambia has revealed that 43% of observed growth faltering was associated with evidence of small intestinal enteropathy.^4^ There are no universally accepted protocols for the diagnosis and treatment of EED. The main impediments are a paucity of validated non-invasive biomarkers of small intestinal health, a lack of understanding of the pathologic state of the small intestine in EED and rudimentary knowledge of disease mechanisms.^5^ While there are several candidate surrogate biomarkers of EED, to our knowledge none of these non-invasive biomarkers have been directly correlated with the diagnostic gold standard: intestinal histopathology. The Bangladesh Environmental Enteric Dysfunction (BEED) study, a community-based intervention study, is designed to validate candidate non-invasive biomarkers of EED and to identify potential therapeutic targets for interventions. This report describes the BEED study design, including the biospecimens and data types that are being collected for analyses.

## Methods and analysis

The protocol for the BEED study (ClinicalTrials.gov identifier NCT02812615) was developed through a collaborative effort among International Centre for Diarrhoeal Disease Research, Bangladesh (icddr,b), Dhaka Medical College and Hospital (DMCH), Bangabandhu Sheikh Mujib Medical University (BSMMU), Dhaka, Dhaka Shishu Hospital, Dhaka, Apollo Hospitals, Dhaka, Bangladesh, University of Virginia, USA and Washington University, USA. The study is funded by the Bill and Melinda Gates Foundation under its Global Health Program (http://www.gatesfoundation.org/How-We-Work/Quick-Links/Grants-Database/Grants/2015/11/OPP1136751). Enrolment has been initiated and is expected to continue until December 2018.

### Objectives

The goals of this study are to (1) develop a histological scoring system for identifying EED and describing its severity; (2) employ this scoring system to test the validity of proposed, candidate non-invasive biomarkers of EED and identify novel biomarkers; (3) test the degree of correlation between EED score and nutritional status defined by anthropometry; (4) determine the role of the gut microbiota in the pathogenesis of EED, in part through transplantation of luminal/mucosal proximal small intestinal and faecal microbiota from malnourished children with and without histopathological evidence of EED into gnotobiotic mice to directly test transmissibility of gut barrier/mucosal immune, growth and metabolic phenotypes and (5) characterise biological pathways that operate in the host and/or gut microbiota that are associated with EED to identify potential therapeutic targets.

### Study settings and participants

**Figure 1:**
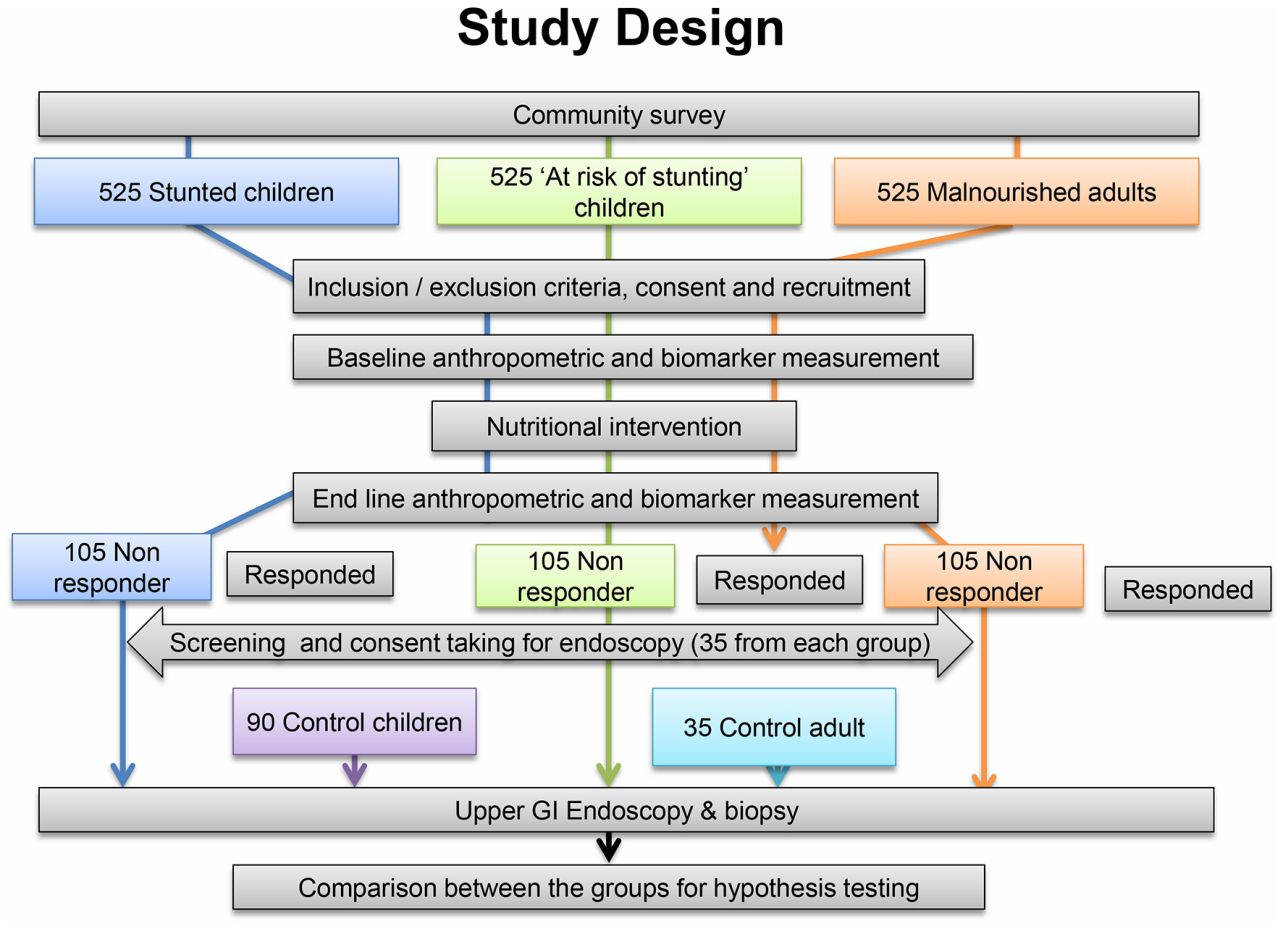
Study design of Bangladesh Environmental Enteric Dysfunction study. GI, gastrointestinal.

The overall design of the BEED study is illustrated in figure 1. There is a community-based nutrition intervention group and a comparison group. In the nutrition intervention group, participants are being recruited from two age groups: a child cohort (age 12–18 months) and an adult cohort (age 18–45 years). The child cohort consists of individuals who are stunted (LAZ <−2) and those who are at risk of stunting (LAZ <−1 to −2). These two subgroups will be used to compare histopathological features of EED with LAZ scores. The adult cohort consists of malnourished individuals (body mass index (BMI) <18.5 kg/m^2^) who will provide an opportunity to further explore the association between body mass (in this case low BMI) and the pathological features of EED and also critically assess the degree to which histopathological features of EED in children manifest in adults.

The BEED study is recruiting two control groups in order to compare the histological features in their duodenal mucosal biopsies with those of documented in cases of EED. Due to obvious ethical considerations, it is not possible to collect small intestinal biopsy samples from entirely healthy and asymptomatic subjects. Therefore, biopsies are being collected from children who are neither stunted nor suffering from EED and who undergo upper gastrointestinal (UGI) endoscopy as part of their clinical care. This control group of children are being recruited by the collaborators at the University of Virginia Hospital in Charlottesville, Virginia, USA. An equal number of age-matched and sex-matched healthy adult controls (BMI >18.5) suffering from functional dyspepsia who have no evidence of any organic diseases are being recruited from the Gastroenterology Outpatient Department of DMCH and the icddr,b Staff Clinic and undergo UGI endoscopy.

Following application of the inclusion/exclusion criteria listed in online supplementary file 1, undernourished children and adults are receiving nutrition and health interventions (table 1). Participants who fail to respond to nutritional therapy are considered eligible for UGI endoscopy with mucosal biopsies. Candidate EED biomarkers are being measured in faeces, urine and plasma samples collected from participants once before and once after the nutritional intervention.

10.1136/bmjopen-2017-017768.supp1 Supplementary data

**Table 1:** Nutrition and health interventions

Intervention	Delivery method
Egg	One egg daily, 6 days a week for either 2 months (children at risk for stunting and malnourished adults) or 3 months (stunted children)
Milk	150 mL whole milk daily, 6 days a week for either 2 months (children at risk for stunting and malnourished adults) or 3 months (stunted children)
Micronutrient sprinkles	–Parents will be given one sachet of multiple micronutrient sprinkles per day to be administered to the child at home with the midday meal for 2 months –Each sachet contains one recommended dietary allowance (RDA) of vitamin A, vitamin C, folic acid, iron and zinc
Nutritional counselling	Parents/caregivers will be provided with nutritional counselling with particular emphasis on adding vegetable oil to the cooked diet as a source of energy as well as sources of animal-based products, such as small fish or chicken meat, and so on along with regular intake of vegetables
Antihelminthic treatment	–As per national guidelines –If participants have not been treated for helminths in the preceding 3 months, will get either 200 mg of albendazole or 10 mg/kg pyrantel pamoate single dose
Treatment	Intercurrent illnesses

The BEED study is conducted among residents of Mirpur, one of the 21 administrative units of the nation’s capital, Dhaka. The Mirpur area is located 7–8 km from the icddr,b Dhaka Hospital at Mohakhali. Mirpur has a population of ~500 000 living in an area of 14.22 km^2^: that is, the population density is more than 38 000 people per square kilometre. The detailed socioeconomic and demographic information of the study location has been published elsewhere.^6^

### Sample size

Participants are being recruited in a cross-sectional manner. Sample size calculations were predicted on the following assumptions: prevalence of stunting at our study site is 40%; after receiving nutrition intervention ~20% will fail to manifest improved linear growth; only one-third will give their consent for endoscopy and 90% of the stunted children will have one or more abnormal gut biomarkers with a precision of 10% and 95% CI. Using the formula n=(Z_1−α/2_)^2^×P×(1−P)/d^2^, where n is the required sample size without attrition; P is the prevalence of one or more abnormal gut biomarker in stunted children=90% or 0.9; d is the level of actual precision=10% or 0.1; Z_1−α/2_ is the value for standard normal distribution at 95% level of significance=1.96, a minimum of 35 stunted children, 35 children at risk of stunting and 35 malnourished adults will be needed for UGI endoscopy and biopsy. Therefore, the final sample size for endoscopy is 105 subjects (35+35+35). As endoscopy is an invasive procedure, we assume that only one-third of those who will fail to respond after nutrition intervention will give consent for the procedure. As a consequence, the ‘basic’ sample size was increased threefold for a minimum of 105 (35×3) stunted children, 105 (35×3) children at risk of stunting and 105 (35×3) malnourished adults. To achieve this goal, 525 stunted children, 525 children at risk of stunting and 525 malnourished adults will be recruited for nutrition interventions, based on the assumption that ~20% of each group will fail to respond despite the nutritional intervention. To enrol the desired numbers of participants, at least 3977 (1312+2665) children 12–18 months old and 2100 adults needed to be surveyed based on the current prevalence of malnutrition in both the age groups. An equal number (35) of age-matched and sex-matched apparently healthy adult controls (BMI >18.5) will be recruited from the outpatient department of DMCH and icddr,b Staff Clinic. Apart from the dyspeptic symptoms by Rome III criteria,^7^ these subjects will have no other symptoms.

### Dietary supplementation

Supplementation (table 1) of the usual home diet with one boiled egg and 150 mL of whole milk provide an additional 745 kilojoules, 11.1 g of protein and 11.5 g of fat to the daily diet of enrolled subjects. The duration of the intervention is 3 months for the stunted cohort and 2 months for those who are at risk for stunting and malnourished adults. Egg and milk were selected considering the nutritional values and the fact that they have shown acceptable to the target population in a small pilot study. Multiple studies have found significant positive effects of egg and milk on linear growth.^8–10^

Nutritional therapy is delivered between the morning and midday meal, anytime between 10:00 am to 11:30 am to avoid the food substitution. Participants are asked to come to the designated nutrition centre daily for their nutritional therapy in order to avoid the issue of food sharing with other family members. A staff member visits the family’s household if a participant defaults in coming to the nutrition centre. Study co-investigators also visit nutrition centres regularly to monitor food left over, absenteeism and other issues of compliance.

We assume that this regimen of food supplementation plus counselling will result in incremental improvement in linear growth of participant children. A participant is discontinued from the study and referred for medical evaluation if he/she shows reluctance to feed for 7 consecutive days, which includes daily intake of ≤50% of the offered food.

### Collection, preparation and archiving of biological samples

All biological samples (blood, urine, stool, breath, duodenal aspirates and endoscopic biopsies) are collected, prepared and preserved (see online supplementary file 2) as per the standard operating procedures (SOPs) prepared for this protocol.

Breath samples are being collected for testing of small intestinal bacterial overgrowth (SIBO). The first SIBO test is performed before the nutrition intervention and a second SIBO test is done 1–2 weeks prior to the endoscopy, so that findings from the endoscopy can be compared with results obtained from the hydrogen breath tests. QuinTron Breath tracker machine and associated QuinTron accessories are used for sample collection and analysis.^11^

Faecal samples are collected and cryopreserved within 20 min of production. At the site of collection, samples are aliquoted into sterile, prelabelled 2 mL cryophials and immediately placed into precharged liquid nitrogen dry cryo-shippers for transport back to the laboratory where they are transferred into a −80°C freezer prior to shipping. No additives, preservatives or media is added to the faecal samples. Culture-independent as well as culture-based analyses of the faecal microbiota (microbial community samples obtained from the UGI tract at the time of endoscopy) will be performed at Washington University in St. Louis, USA.

All other samples for biomarker assessment are carried to the laboratory using cool box and cool packs. For blood samples, with all aseptic precautions 5 mL venous blood is collected in a blood collection tube (S-Monovette 7.5 mL, Sarstedt) and transported to laboratory. Initially, 0.5 mL blood is transferred into a centrifuge tube and the remaining 4.5 mL is centrifuged for the separation of plasma. The plasma is then aliquoted and stored at −80°C. Chlorhexidine is added to urine samples as a preservative. All the samples from each individual are collected within a 3–4 days window period.

### Biomarkers

A wide array of biomarkers of enteropathy will be tested in the BEED study. A list of candidate biomarkers was prepared after a thorough literature review and analysis of data emanating from the Etiology, Risk Factors and Interactions of Enteric Infections and Malnutrition and the Consequences for Child Health and Development (MAL-ED)^12–14^ and Performance of Rotavirus and Oral Polio Vaccines in Developing Countries (PROVIDE)^15^ studies (table 2).

**Table 2:** Summary table for non-invasive biomarker candidates and related processes

Process of gut health	Biospecimen	Non-invasive biomarkers
Epithelial health and repair	Faeces	Reg1B
Intestinal barrier dysfunction and bacterial translocation	Blood and urine	Alpha-1-antitrypsin, lactulose/rhamnose ratio
Intestinal inflammation	Faeces	Myeloperoxidase, neopterin, calprotectin
Systemic inflammation	Blood	sCD14, CRP, AGP, KT ratio
Epigenetic metabolomes	Blood	LRP1
Enteric infection	Faeces, breath sample	TaqMan Array Card, SIBO
Nutrient malabsorption	Blood	Ferritin, zinc, GLP-2, pepsinogen I/II ratio
Celiac Disease screening	Blood	IgA tissue transglutaminase, total IgA

AGP, alpha 1-glycoprotein; CRP, C reactive protein; KT ratio, kynurenine/tryptophan ratio; LRP1, low density lipoprotein receptor-related protein 1; GLP-2, glucagon-like peptide-2; Reg1B, regenerating family member 1 beta proteins; sCD14, soluble cluster of differentiation 14; SIBO, small intestinal bacterial overgrowth.

### Indications for and details of the procedures used for UGI tract endoscopy and mucosal biopsy

After completion of nutritional therapy, all enrolled participants are assessed for responses. Definitions of ‘failure to thrive/respond’ are as follows:

- LAZ score remains <−2 for members of the stunted children cohort
- LAZ score remains <−1 for the ‘at risk of stunting’ children cohort
- BMI <18.5 kg/m^2^ and <10% increase in BMI for those in the adult cohort

Participants who meet the study definition of ‘failure to thrive/respond’ are further checked for the presence of any secondary cause of malnutrition such as tuberculosis, any parasitic infection, and so on. If nothing conclusive is found, the participant may undergo preparations for UGI endoscopy with biopsy. Participants with celiac disease (as defined by tissue transglutaminase IgA, and clotting disorders (determined by prothrombin time/International normalised ratio (INR) coagulopathy tests) will be excluded from the study. There is no published information about the prevalence of celiac disease in Bangladesh from any representative sample.^16^ One study of hospitalised adult patients with irritable bowel syndrome observed that the prevalence of celiac disease was 9%.^17^ In the BEED study, immediate test results for celiac disease are not available; therefore, we are unable to screen out individuals with this disorder before nutrition intervention is initiated. Nonetheless, BEED will screen 1575 participants for celiac disease; as such, it will be the first study in Bangladesh to explore the prevalence of this disorder in a paediatric population. Necessary management and counselling is provided to the participants suffering from celiac disease or clotting disorders.

Endoscopy of the UGI tract is performed as per standards recommended by North American Society for Pediatric Gastroenterology, Hepatology and Nutrition, the American College of Gastroenterology and the American Society for Gastrointestinal Endoscopy (ASGE).^18^ The procedure is generally scheduled in the early morning after fasting from midnight. Gastroenterologists, who perform endoscopy, discuss with the subjects and their guardians regarding the indication of the procedure, the sedation plan, the risks associated with sedation and describe the total procedure, likely benefits, common adverse events (AEs), alternatives to the procedure and the prognosis of the subjects if endoscopy is declined. After this discussion and proper counselling, the gastroenterologists personally obtain consent from the subjects on the day of the procedure. A separate consent is obtained for sedation. A preprocedure assessment that includes medical history and physical examination is also completed by the gastroenterologist himself.

Once the preprocedure assessment is completed and no concerns are identified by the gastroenterologist, the subject is proceeded to the endoscopy room. A qualified anaesthetist attends the procedure to assess and administer the steps, as required, for sedation. Before sedation is administered, the intended level of sedation is graded (nil, minimal, moderate and deep sedation or general anaesthesia) as per the recent guidelines of ASGE.^18 19^ Children are sedated using general anaesthesia by an expert and qualified anaesthetist. UGI endoscopies of adult participants are performed under conscious sedation using midazolam. The oropharynx is anaesthetised with 10% lidocaine spray; concomitantly, midazolam (3–5 mg) is injected intravenously to induce conscious sedation.^19^

The endoscopic procedure is performed with a goal of collecting up to six biopsies from each child and up to eight biopsy samples from each adult participant. As per the protocol, one biopsy is taken from the duodenal bulb; the rest of the biopsies are collected from the distal part of the duodenum. Duodenal fluid is aspirated for culture-independent and culture-based assessments of the composition of the proximal small intestinal microbial community and for assays of host and microbial products. Secretions are rapidly aspirated first from the second portion of the duodenum using sterile Endoscopic Retrograde Cholangiopancreatography (ERCP) catheters. Following this initial aspiration, the postbulbar mucosa is irrigated with a maximum of 25 mL of sterile 0.9% normal saline, and the lumen contents are aspirated through the ERCP catheter. The lavaged aspirates are aliquoted and processed as per the SOP described in table 3.

**Table 3:** Biopsy specimen processing

Specimen	Recipient fluid	Processing	Purpose
Biopsy #1	RNAlater	Nucleic acid extraction	Transcriptomics
Biopsy #2	HBSS	FACS	Cell isolation
Biopsy #3	None	Flash frozen	Culture-independent and culture-based assays of mucosa-associated microbial community
Biopsy #4	None	Flash frozen	Archiving for validation of drug targets
Biopsies #5 and #6*	Formalin	Paraffin embedded	Histochemistry (H&E) and immunohistochemistry; Morphometric analyses and EED scoring
Duodenal Aspirate	Microbiota: glycerol/cysteine solution Other: Nil	Precharged N_2_ dry shipper to −80 freezer	Culture-independent and culture-based assays

*Biopsy site: biopsy #6 from duodenal bulb, all others distal to the ampulla of Vater.

EED, environmental enteric dysfunction; FACS, fluorescence-activated cell sorting; HBSS, Hank’s balanced salt solution.

Participant undergoing endoscopy is observed in the postprocedure recovery room until he/she meets predetermined standard discharge criteria.^20^ The subjects who undergo the endoscopy procedure are followed up for 14 days after the procedure to determine whether any AEs have occurred after discharging from the endoscopy unit and whether these are attributable to the procedure. An appropriate course of action will be followed if such events occur; the cost of management of all postprocedure complications will be borne by the study. Participants receive appropriate treatment based on the results of reports from the endoscopy and biopsy procedure. As for example, participants diagnosed endoscopically with antral gastritis are screened for *Helicobacter pylori* and prescribed proper treatment, as per standard recommendations.

### Protocol timeline

The overall schedule for enrolment, interventions and assessments, including UGI endoscopy and biopsies, is described in table 4.

**Table 4:** Schedule of enrolment, interventions and assessments

	Study period							
	Enrolment	Preintervention	Nutrition intervention			Postintervention assessment	Eligibility assessment for UGI endoscopy	UGI endoscopy and biopsy
Timepoint	***−T_1_***	0	Month 1 (***T_1_***)	Month 2 (***T_2_***)	Month 3 (***T_3_***)	Week 1 (***T_4_***)	Week 2 (***T_5_***)	Week 3 (***T_6_***)
Enrolment	X							
Pre-enrolment survey	X							
Eligibility screen	X							
Informed consent	X						X	
Interventions								
Stunted children			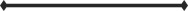					
Children at risk of stunting			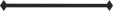					
Malnourished adult			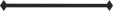					
Assessments								
SES, WASH, FFQ		X				X		
Anthropometry*		X	X	X	X	X		
Blood biomarkers		X				X		
Stool biomarkers and TAC		X				X		
Microbiota		X				X		
Urine L:R		X				X		
Breath SIBO		X				X		
UGI endoscopy and biopsy		X				X		
Histopathology, IHC, morphometry								X

*Anthropometry done fortnightly.

IHC, immunohistochemistry; L:R, lactulose/rhamnose; SIBO, small intestinal bacterial overgrowth; TAC, TaqMan Array Card; UGI, upper gastrointestinal; SES, socio-economic status; WASH, water-sanitation-hygiene; FFQ, food frequency questionnaire.

### Data collection, management and storage

Data collection tools for this study include case report forms, laboratory worksheets and source documentation. Complete source documentation (study visits, laboratory reports, and so on) is done for each participant in individual study charts. All laboratory specimens, reports, study data collection and administrative forms are identified by coded number to maintain participant confidentiality and to enable tracking throughout the study. Bar-coded labels are used for all laboratory specimens. All information regarding study subjects are kept in password-protected computer files, or in locked file cabinets that can be accessed only by authorised study personnel. Anthropometric data (table 4) are collected twice in a month. The total amounts of food offered, consumed and leftover are recorded. All foods are weighed with precision scales (see online supplementary file 3). Multiple food frequency data are collected from all the participants, whereas a 24 hours dietary recall data are being collected from a subgroup of randomly selected children as well as adults (50 in each group) to assess their food habits and food substitution, if any.

### Data analysis plan

This study will yield a rich dataset containing socio-demographic and anthropometric information, food security status, plus faecal, urine, plasma and breath associated biomarkers, the results of histopathological measurements as well as data related to small intestinal gut health risk factors. After evaluation of biopsy specimens, a pathologist will assign a histopathological grade, in a blinded fashion, based on histological changes deemed to be signatures of EED: these include changes in mucosal architecture (shortening of the villi, increase in the depth of the crypts), changes in immune-inflammatory cell content (lymphocytic infiltration into the lamina propria and epithelium) and changes in epithelial features (goblet cell content, evidence of enterocyte injury and so on).^21^ Statistical tests of correlations between histopathological scores, biomarker levels and anthropometric changes will be performed. An ‘inter-group’ comparison (stunted children vs children at risk of stunting; stunted vs non-stunted, and so on) will be performed for validation of histopathological grading. The analysis plan includes relatively simple linear modelling of all continuous and dichotomous measures to establish whether biomarkers are associated with anthropological changes or not. Biomarkers exhibiting statistically significant changes will be identified and their final validation will be performed by comparing their levels with histopathological changes.

An important research question is whether enteric infections, and gut immune responses to those infections, are associated. While preliminary inferences of linkage are possible from a careful interpretation of simple univariate and bivariate analyses, instrumental variable analysis (for the control of unmeasured covariates) will also be performed.

Being an invasive procedure requiring hospitalisation, histopathological diagnosis of EED will not be possible as a routine approach in at-risk populations in low-income settings. A goal of this project is to validate surrogate biomarkers of the disease and use these for developing models to predict future negative histopathological and clinical outcomes. From a list of significant biomarker variables, we will select a ‘best’ subset by using a branch and bound algorithm. This method will test all biomarker subsets in contrast to the more usual forward or reverse stepwise regression. As development of prediction model risks over-fitting to the data set, we plan to partition the data set into model training (2/3) and test (1/3) subsets, using the test set at the end of the fitting to establish an unbiased estimate of the generalisation error. We will estimate the prediction error in the training set using cross-validation methods. A subset model of biomarkers will also be used to create a composite score of EED.

### Monitoring and quality control measures

We are using previously established and validated SOPs for data collection, monitoring and quality control measures.^12 22^ A field supervisor re-collects 10% of all collected data, including anthropometry and dietary assessments. Monitoring reports are prepared weekly. An Excel-based program is used for scheduling data and sample collection from each participant. All activities of the BEED study are being performed according to a Manual of Procedures (a collection of all SOPs). A summary of quality control measures is described in online supplementary file 4.

### Challenges

Several questions arose in the development and implementation of the BEED study, all of which were brought to the Investigators’ Committee for discussion and resolution. For example, AEs that may arise during endoscopy were a big concern and to solve the issues, investigators have developed a priori definitions, assessment criteria and action guidelines. Morbidity and poor appetite also represent a big challenge; by providing proper counselling and by practising supportive feeding techniques, this issue can be resolved.

## Ethics and dissemination

### Ethical approvals

Ethical approvals were obtained from Research Review Committee (RRC) and Ethical Review Committee (ERC) of icddr,b (protocol no: PR-16007; Version 1.03; March 1, 2016), the Ethical Committee of Dhaka Medical College (DMC/ ECC/2016/39). Institutional Review Board for Health Sciences Research (IRB-HSR) of University of Virginia, Charlottesville, the Human Research Protection Office (HRPO) of Washington University in St. Louis (the latter for analyses of collected biospecimens).

### Consent

Each participant enrolled in BEED study is treated according to what is morally right and proper. Separate consent forms are being used for children and adult participants. After complete disclosure, a signed informed consent statement is obtained from each subject. For minors, informed consent is obtained from the parents or authorised legal guardian of the subject. The consenting process takes place, preferably, in the residence of the subject. If the subject or the parent(s)/guardian agree to participate in the study, they sign the consent form or provide an impression of their left thumb. The investigator and a witness also sign the form. For endoscopy, a separate consent form is used and the aforementioned procedure is followed. The consent form for endoscopy clearly and fully describes, and demystifies, all aspects of the process, including the risks related with the procedure. No information is remained withheld from the participant.

### Adverse events

Expected AEs for this protocol are those related to the endoscopy/biopsy procedure that do not qualify as a serious adverse event (SAE) and those associated with phlebotomy and ingestion of lactulose/rhamnose solution. Both serious and non-SAEs are assessed for their severity, their relationship to study participation and the actions taken and their outcomes. All SAEs are being reported to the ERC of icddr,b within 24 hours of the site’s awareness of the event. In the event that medical care is required outside of the protocol, all necessary and available treatments are provided, free of cost.

### Safety issues for UGI endoscopy and biopsy

Although never without elements of risk during the procedure and anaesthesia, endoscopy is a safe procedure when conducted by trained experienced personnel in a well-equipped facility. In Gambia, a study was done where 40 children were recruited and underwent endoscopy under intravenous sedation.^23^ In the University Teaching Hospital, Lusaka, Zambia, investigators collected duodenal biopsy samples via endoscopy under sedation with midazolam from 41 malnourished children to compare their inflammatory status with normal children.^24^ Neither of these studies reported any endoscopy-related AEs during or after the procedures. Following standard recommendations and guidelines, endoscopic procedures are performed by members of a team of co-investigators consisting of experienced consultant gastroenterologists from BSMMU, DMCH, Apollo Hospital, Dhaka and icddr,b. A qualified anaesthetist attends the procedure to assess and administer steps as required for sedation. The participant’s oxygen saturation level, pulse rate and blood pressure are continuously monitored throughout the procedure. Resuscitation measures and complete ICU support remain available during the procedure for immediate resuscitation if necessary. Clinical findings from the biopsies (eg, presence of gastritis) are made available quickly so that appropriate treatment can be undertaken in a timely manner.

### Dissemination and publication

The data, results and other findings resulting from this study will be published only after approval by a committee consisting of the investigators of the protocol. The International Committee of Medical Journal Editors guidelines will be used to establish authorship on papers.

#### Project status

As of May 2017, participant enrolment is ongoing.

## Supplementary Material

Reviewer comments

Author's manuscript
