# Supplementary material for: Bangladesh Environmental Enteric Dysfunction (BEED) study: protocol for a community-based intervention study to validate non-invasive biomarkers of environmental enteric dysfunction
Source: BMJ Open. 2017 Aug 11;7(8):e017768. doi: 10.1136/bmjopen-2017-017768 (PMC5724211; doi:10.1136/bmjopen-2017-017768)
Supplement: Supplementary data [file bmjopen-2017-017768supp001.pdf]

SUPPLEMENTARY FILE 1

Inclusion and exclusion criteria

**Inclusion criteria**	**Exclusion criteria**
Child cohort	
- Parent(s) willing to sign consent form<br>- Child age 12-18 months<br>- LAZ < -2 (for stunted cohort) or<br>- LAZ between < -1 to -2 (for 'at risk of stunting' cohort)<br>- Parent(s) willing to bring child to the study site for nutritional therapy<br>- Parents willing to have child undergo laboratory investigations and upper GI endoscopy and biopsy if the child fails to respond to nutritional therapy	- Severe acute malnutrition (SAM), severe anemia (<8 g hemoglobin/dL), TB, other chronic diseases or any congenital disorder or deformity<br>- Diarrhoea: Ongoing episode of diarrhoea, history of persistent diarrhoea in the past month or history of acute diarrhoea in the past 7 days<br>- Known allergy to eggs or milk or milk intolerance
Adult cohort	
- Willing to sign consent form<br>- Age 18-45 years<br>- BMI < 18.5<br>- Willing to visit the study site every day for 2 months for nutritional therapy<br>- Willing to undergo endoscopy and biopsy if he/she fails to respond to nutritional therapy	- Severe anemia (<8 g/dl), TB and other chronic diseases<br>- Pregnant women, lactating women, drug abuse, known psychiatric disorders<br>- High clinical suspicion of cancer or other chronic or acute diseases<br>- Known allergy to any components of the nutrition intervention.

SUPPLEMENTARY FILE 2

Biological samples aliquoting and preservation

Sample	Amount	Aliquots	Copy 1	Copy 2	Copy 3	Copy 4	Copy 5	Copy 6	Preserve
Blood	5 mL	6	Whole blood	1 mL plasma Biomarkers	1 mL plasma Biomarkers	200 µL Plasma KT	1 mL plasma Storage	Whole blood Cell	-80°C
Feces	2 g	2	Microbiota	Microbiota	-	-	-	-	-80°C
Feces	15-20 g	5	Biomarker	Biomarker	TAC	Storage	Storage	-	-80°C
Urine	10 mL	3	1 mL LR	1 mL Storage	1 mL Storage	-	-	-	-80°C

SUPPLEMENTARY FILE 3

Anthropometric data and food collection tools

**Anthropometric scale**					
Child cohort	Instrument	Precision	Adult cohort	Instrument	Precision
Weight	Seca digital weighing scale (http://meenamedical.com/uploads/Manuals/Scale/seca_727_Brochure.pdf)	1 gm	Weight	Tanita step-on type weighing scale (http://healthmonitor.xyz/B0052BPUCA-health-meter-digital-hd-661-wh-white-step-on-type-switch-to-turn-the-ride-tanita)	100gm
Length	Seca Infantometer (http://www.seca.com/en_ae/products/all-products/product-details/seca416.html)	0.1 cm	Height	Seca Stable stadiometer for mobile height measurement (http://www.seca.com/en_ae/products/all-products/product-details/seca217.html)	0.1 cm
Triceps skinfold thickness	Harpenden Skin fold callipers (http://www.harpenden-skinfold.com/)	99%	Triceps skinfold thickness	Harpenden Skin fold callipers (http://www.harpenden-skinfold.com/)	99%
Head Circumference	Measuring tape				
Mid upper Arm Circumference	MUAC tape				

**Measuring scale**		
Name	Purpose	Precision
Sartorius TE4101 digital scale	Measurement of egg http://scaleman.com/sartorius-talent-digital-te4101-compact-scale.html	0.1 g
KERN EMB 200-2 Precision balance	Sugar measurement https://www.kern-sohn.com/en/EMB	0.01g
PYREX™ VISTA™ Griffin Beakers (250 ml)	For measuring milk during offering https://www.fishersci.com/shop/products/pyrex-vista-griffin-beakers-250ml/07250056	

SUPPLEMENTARY FILE 4

Quality Controls

Anthropometric data	• All scales are calibrated daily<br>• 3 measurement are taken and the average value is recorded<br>• 10% data are collected by the supervisor
Food scales	• All scales are calibrated daily<br>• Same cup is used for measurement
Data entry	• Double data entry system is used<br>• Interrater reliability (kappa) is a measured to examine the agreement
Egg-Milk quality control	• Assessment of nutrition content- thrice in a year<br>• Assessment for salmonella/avian influenza - thrice in a year
