# Supplementary material for: Bangladesh Environmental Enteric Dysfunction (BEED) study: protocol for a community-based intervention study to validate non-invasive biomarkers of environmental enteric dysfunction
Source: BMJ Open. 2017 Aug 11;7(8):e017768. doi: 10.1136/bmjopen-2017-017768 (PMC5724211; doi:10.1136/bmjopen-2017-017768)
Supplement: Author's manuscript [file bmjopen-2017-017768.draft_revisions.pdf]

BMJ Open is committed to open peer review. As part of this commitment we make the peer review history of every article we publish publicly available.

When an article is published we post the peer reviewers' comments and the authors' responses online. We also post the versions of the paper that were used during peer review. These are the versions that the peer review comments apply to.

The versions of the paper that follow are the versions that were submitted during the peer review process. They are not the versions of record or the final published versions. They should not be cited or distributed as the published version of this manuscript.

BMJ Open is an open access journal and the full, final, typeset and author-corrected version of record of the manuscript is available on our site with no access controls, subscription charges or pay-per-view fees (http://bmjopen.bmj.com).

If you have any questions on BMJ Open's open peer review process please email editorial.bmjopen@bmj.com

BMJ Open

Systematic Review of preoperative physical activity and its impact on post-cardiac surgical outcomes

Journal:	*BMJ Open*
Manuscript ID	bmjopen-2016-015712
Article Type:	Research
Date Submitted by the Author:	22-Dec-2016
Complete List of Authors:	Kehler, D. Scott<br>Stammers, Andrew N.; University of Manitoba,<br>Tangri, Navdeep; University of Manitoba, Medicine<br>Hiebert, Brett<br>Fransoo, Randy; University of Manitoba, Community Health Sciences<br>Schultz, Annette; University of Manitoba, Nursing<br>Macdonald, Kerry<br>Giacomontonio, Nicholas<br>Hassan, Ansar; New Brunswick Heart Centre,<br>Légaré, Jean-Francois<br>Arora, Rakesh C.; University of Manitoba,<br>Duhamel, Todd A.; University of Manitoba,
**Primary Subject Heading**:	Cardiovascular medicine
Secondary Subject Heading:	Rehabilitation medicine
Keywords:	Cardiac surgery < SURGERY, REHABILITATION MEDICINE, Exercise, Postoperative complications, Prognosis

SCHOLARONE™
Manuscripts

Title: Systematic Review of preoperative physical activity and its impact on post-cardiac surgical outcomes

Authors

D. Scott Kehler, MSc,§[1,2] Andrew N. Stammers, B. Kin,[1,2] Navdeep Tangri, PhD,[3] Brett Hiebert, MSc,[4] Randy Fransoo, PhD,[5] Annette S. Schultz, RN, PhD,[6] Kerry Macdonald, MLIS,[7] Nicholas Giacomantonio, MD, FRCPC,[8] Ansar Hassan, MD, PhD, FRCSC,[9] Jean-Francois Légaré, MD, FRCSC,[10] Rakesh C. Arora, MD, PhD, FRCSC, FACS,†[4] and Todd A. Duhamel, PhD.†[1,2]

†Contributed equally as senior authors

§Corresponding author

Affiliations

[1]Health, Leisure & Human Performance Research Institute, Faculty of Kinesiology and Recreation Management, University of Manitoba, Winnipeg, Canada;

[2]Institute of Cardiovascular Sciences, St. Boniface Hospital Research Centre, Winnipeg, Canada;

[3]Seven Oaks Hospital Research Centre, Winnipeg, Canada;

[4]Department of Surgery, University of Manitoba and Cardiac Sciences Program, Winnipeg, Canada;

[5]Department of Community Health Sciences and Manitoba Centre for Health Policy, University of Manitoba, Winnipeg, Canada;

[6]College of Nursing, Faculty of Heath Sciences, University of Manitoba Winnipeg, Canada;

[7]Seven Oaks Hospital Library, Winnipeg, Canada;

[8]Division of Cardiology, Department of Medicine, Dalhousie University, Halifax, Canada;

[9]Department of Cardiac Surgery, New Brunswick Heart Centre, Saint John Regional Hospital, Saint John, Canada;

[10]Division of Cardiac Surgery, Department of Surgery, Dalhousie University, Halifax, Canada;

For peer review only

Address for correspondence:

1. Mr. Scott Kehler, 212 Active Living Centre, Winnipeg, MB, Canada, R3T 2N2

Telephone +1204 918 9131 Fax +1204 474 7634

e-mail: skehler@sbrc.ca

Acknowledgements:

Dr. Francesco Cacciatore and his research group provided additional data on ICU length of stay. TAD was supported by a Manitoba Health Research Council (MHRC) Establishment Grant. DSK was supported by an MHRC Studentship, A CIHR Frederick Banting and Charles Best Canada Graduate Scholarship, a CIHR Strategic Training Initiative in Health Research (STIHR) Knowledge Translation Canada Student Fellowship, a CIHR STIHR Population Intervention for Chronic Disease Prevention Fellowship and the Heart and Stroke Foundation Dr. Dexter Harvey Award. ANS was supported by a CIHR Canada Graduate Scholarship.

Authors' Contributions:

DSK was responsible for 1) analysis and interpretation of data, 2) drafting and revising the manuscript, and 3) consenting for manuscript submission. ANS, BH, NT, RF, ASH, NG, AH, JL were responsible for 1) analysis and interpretation of data, 2) revising the manuscript, and 3) consenting for manuscript submission. KM was responsible for 1) developing the systematic review literature search, 2) analysis and interpretation of data, 3) drafting and revising the manuscript, and 3) consenting for manuscript submission. RCA and TAD were responsible for 1) the conception and design, and analysis and interpretation of data, 2) revising the manuscript, and 3) consenting for manuscript submission RCA and TAD are co-senior authors.

Funding:

The work was supported by an Operating Grant from the Canadian Institutes of Health Research

For peer review only

grant number 326290.

Ethics Approval:

Not applicable

Systematic review registration number:

PROSPERO: CRD42015023606

Competing Interests:

The authors have no conflicts of interest to declare

Word count (not including abstract, tables, and references): 2956

Figures and tables: 5

For peer review only

ABSTRACT:

Objectives: The objective of this systematic review was to study the impact of preoperative physical activity levels adult cardiac surgical patients' postoperative: 1) major adverse cardiac and cerebrovascular events (MACCE), 2) adverse events within 30 days, 3) hospital length of stay (HLOS), 4) intensive care unit length of stay (ICU LOS), 5) activities of daily living (ADLs), 6) quality of life, 7) cardiac rehabilitation attendance, and 8) physical activity behavior.

Methods: A systematic search of MEDLINE, Embase, AgeLine, and Cochrane library for cohort studies was conducted.

Results: Eleven studies (n=5,733 patients) met the inclusion criteria. Only self-reported physical activity tools were used. Few studies used multivariate analyses to compare active versus inactive patients prior to surgery. When comparing patients who were active versus inactive preoperatively, there were mixed findings for MACCE, 30 day adverse events, HLOS, and ICU LOS. Of the studies which adjusted for confounding variables, five studies found a protective, independent association between physical activity and MACCE (n= 1), 30 day postoperative events (n= 2), hospital length of stay (n= 1), and ICU length of stay (n= 1), but two studies found no protective association for 30 day postoperative events (n= 1) and postoperative ADLs (n= 1). No studies investigated if activity status before surgery impacted quality of life or cardiac rehabilitation attendance postoperatively. Three studies found that active patients prior to surgery were more likely to be inactive postoperatively.

Conclusion: Due to the mixed findings, the literature does not presently support that self-reported preoperative physical activity behavior is associated with postoperative cardiac-surgical outcomes. Future studies should objectively measure physical activity, clearly define outcomes, and adjust for clinically relevant variables.

Registration: PROSPERO number CRD42015023606.

Keywords: Cardiac Surgical Procedures, Exercise, Prognosis, Postoperative Complications

For peer review only

ARTICLE SUMMARY

Strengths and limitations of this study

- There were mixed findings regarding the impact of physical activity on post cardiac-surgical outcomes.
- Only self-reported physical activity tools were used.
- The multiple tools to measure physical activity and the variety of definitions of outcomes did not allow for a quantitative synthesis (meta-analysis).

For peer review only

INTRODUCTION

Recent reports suggest that more than half of cardiac surgeries are being performed on older adults who are more likely to be frail and have multiple co-morbidities.[1] While cardiac surgery has been shown to improve the outcomes of these patients, more than 75% of major perioperative complications and deaths occur in older adults.[2,3] Before surgery, many of these patients are de-conditioned and have diminished resilience in the face of major stressors such as cardiac surgery, and it has been postulated that they could benefit from a therapeutic intervention prior to their major surgical procedure in order to reduce their operative risk. However, little information exists to evaluate the benefit of preoperative risk reduction strategies for the older cardiac surgery patient.

Adopting and sustaining a more physically active lifestyle is typically intended to be a part of an interdisciplinary rehabilitation plan that is instituted postoperatively and has been shown to reduce the risk of cardiac mortality and hospital admissions and improve health-related (QOL) in patients.[4] Importantly, older adults who sustain a physically active lifestyle after a postoperatively exercise-based rehabilitation program can continue to improve their functional walking status.[5] However, evidence suggests that cardiac surgery patients are highly sedentary during the preoperative period, especially in older adults.[6] Furthermore, few randomized controlled trials exist which evaluate the therapeutic benefit of preoperative lifestyle modification in patients undergoing cardiac surgery.[7–9] Information regarding the link between preoperative physical activity and postoperative health outcomes in cardiac surgery patients would be valuable for healthcare providers to assist them in selecting patients who might benefit from preoperative exercise therapy.

The purpose of this systematic review was to compare the following postoperative outcomes between cardiac surgery patients defined as physically active prior to surgery and those who were defined as physically inactive preoperatively: 1) major adverse cerebrovascular and cardiovascular events (MACCE) 2) 30-day adverse events 3) hospital length of stay, 4) Intensive Care Unit (ICU) length of stay, 5) health-

For peer review only

related QOL, 6) activities of daily living (ADL) 7) cardiac rehabilitation attendance and 8) physical activity levels postoperatively.

For peer review only

MATERIAL AND METHODS

The protocol for this systematic review has been described in PROSPERO: CRD42015023606. Note the following *ad-hoc* changes to the previous protocol: ICU length of stay and postoperative physical activity as additional outcomes were explored in this systematic review.

Eligibility criteria

Eligible studies included cohort studies which examined adult (>18 years) cardiac surgery patients undergoing coronary artery bypass grafting (CABG), aortic or mitral valve repair/replacement, transcatheter aortic valve implantation, or combined procedures. Studies with patients undergoing congenital cardiac surgery, heart transplantation or left ventricular assist device implantation procedures were excluded. Studies could compare physically active versus inactive patients prior to cardiac surgery on the basis of subjective (e.g., questionnaire) or objective (e.g., pedometer, accelerometry) assessments of physical activity.

Eligible studies had to compare at least one of the following postoperative outcomes: MACCE defined as death, stroke, myocardial infarction, and the need for emergency cardiac surgery; 30-day adverse events as defined by the Society of Thoracic Surgeons (STS);[10] Hospital length of stay; ICU length of stay; health-related QOL with any assessment tool; ADLs using any evaluation strategy; cardiac rehabilitation attendance; and physical activity behavior using either subjective or objective forms of assessment.

For peer review only

Search strategy

The search strategy was completed by a librarian and reviewed by a second librarian. The search included keywords and controlled vocabulary. English language limits were applied. Databases used included MEDLINE, Embase, AgeLine, and Cochrane Library (CDSR, CENTRAL, DARE). The MEDLINE strategy was registered and published online in PROSPERO (http://www.crd.york.ac.uk/PROSPEROFILES/23606_STRATEGY_20150518.pdf). The search was validated through a cross-check of references of studies selected for inclusion. In addition, conference abstracts were hand searched using the Internet. Attempts were made to contact authors of conference abstracts to determine if their findings were published in a peer-reviewed journal.

Study selection

The title, abstract and full-text article screening processes were independently completed by two reviewers. A training exercise for the title and abstract phase was conducted by the independent reviewers using a random sample of 100 titles and abstracts. Discrepancies in studies for inclusion were resolved by discussion of the two reviewers. The final observed agreement was 98% with a kappa statistic of 0.47 for the title and abstract screen. One training exercise of 10 randomly selected articles was completed for the full-text screen. Discrepancies for inclusion were resolved through discussion. The observed agreement for the full-text screen was 96% with a kappa statistic of 0.83.

Data abstraction

Two reviewers independently extracted relevant data for the selected outcomes described above. Discrepancies in the data extraction procedure were resolved through discussion. Data abstraction items

For peer review only

included study characteristics (e.g., authors, year of publication, sample size, follow up time points if relevant), patient characteristics (e.g., age, sex, surgery type), physical activity tool used, and the outcomes which were measured.

Risk of bias assessment

Two reviewers independently reviewed the risk of bias of each included study using the Newcastle-Ottawa Scale.[11] Items within this tool assess the risk of bias associated with selection of participants, comparability (e.g., study authors controlled for patient demographics and clinical characteristics), and outcome assessment (e.g., data collection method for outcome, sufficient follow-up, and adequacy of follow up of cohorts). Each study was given a score within each category (Selection: 0-4; Comparability: 0-2; and Outcome: 0-3) and an overall score ranging from 0-9. A score of zero suggests an increased risk of bias and a higher score suggests a lower risk of bias.

Quantitative synthesis

Due to the significant heterogeneity between studies in terms of physical activity assessment tools used and outcomes assessed, meta-analyses were not performed.

For peer review only

RESULTS

The literature search results are shown in Figure 1. After removing duplicates, 5722 articles were title and abstract screened. A total of 137 articles were then assessed in full-text. Eleven studies met the eligibility criteria for the final analysis, and they included a total of 5,733 patients.[12–22]

An overview of the included studies can be viewed in Table 1. Five studies evaluated CABG only patients,[12–16] four evaluated both CABG and/or valve procedures,[17–21] and one study evaluated isolated aortic valve repair patients.[22] The average age of participants in different studies ranged from 60 years[15,16] to 75 years.[17,18,21,22] Six studies excluded patients with physical impairments or with New York Heart Association heart failure class IV symptoms (severe cardiac symptoms)[14–19] but in general exclusion criteria were not explicitly reported. Studies were conducted in the Netherlands,[12,14,20,22] Brazil,[13,15,16] Italy,[17–19] and the United States.[21] Two studies used the same patient sample, but examined different outcomes.[15,16] The sample size of studies ranged from 35[21] to 3150.[20]

The physical activity assessments in each study were based on self-reported assessment tools. Four studies used the Corpus Christi Heart Project questionnaire;[12,14,20,22] three studies used a structured questionnaire confirmed by the Minnesota Leisure Time Physical Activity Questionnaire[13] or the Baecke Usual Physical Activity questionnaire;[15,16] two studies used the Physical Activity Scale for the Elderly;[17,19] one study used the Harvard Alumni Questionnaire;[18] and one study used The Health and Retirement Survey physical activity-related questions.[21]

For peer review only

MACCE

Outcomes within the definition of MACCE were evaluated in four studies (Table 2).[13,15,17,22] The follow-up periods were one,[13] two, [15,22] and five years[17] postoperatively. Unadjusted differences between active versus inactive patients and MACCE (defined as atrial fibrillation, hospital admission, reoperation and MI) were found one-year postoperatively in one study.[13] Another study found no differences (defined as mortality, re-hospitalization, cerebrovascular accident and MI) at two years postoperatively.[15] One study found that unadjusted rates of mortality within two years post-surgery was significantly higher in the active versus inactive group.[22] One study found a significant and dose-response relationship between physical activity and postoperative cardiac and all-cause mortality after controlling for preoperative demographics, medical history, medications, and clinical characteristics.[17]

30-day events

Five studies evaluated postoperative events within 30 days of surgery (Table 2).[12,16–18,20] The postoperative events measured varied significantly between the studies. Three studies examined if physical activity was an independent protective factor against postoperative events. [16,18,20] Physical activity was an independent protective factor against the combined outcome of mortality, MI, and reoperation;[16] as well as postoperative atrial fibrillation;[18] but not for in-hospital or 30-day mortality.[20]

Postoperative health-related QOL

No studies evaluated postoperative health-related QOL.

Hospital and ICU length of stay

For peer review only

Three studies compared hospital length of stay between active vs. inactive cardiac surgery patients (Table 3).[12,13,16] Hospital length of stay was longer in the inactive group in two of three studies. One study did not report hospital length of stay summary statistics between the active vs. inactive groups.[16] However that study reported an independent association between the preoperative active vs. inactive group and a reduced likelihood of prolonged hospital length of stay, though "prolonged" was not defined in the study.

Three studies compared ICU length of stay between the preoperative physical activity groups (Table 3).[12,19,20] Two of three studies found that the inactive group had a significantly longer ICU length of stay compared to the active group.[12,20] One study conducted a multivariate analysis, and found that the active group was less likely to have a prolonged ICU length of stay >3 days compared to the inactive group after controlling for age, off-pump CABG, stroke, and renal failure.[19]

Postoperative ADLs

One study examined the impact of preoperative physical activity and postoperative ADLs at the time of hospital discharge and revealed no statistically significant ($p = 0.079$) association between the two after adjusting for preoperative demographics and clinical variables.[19]

Cardiac rehabilitation attendance

No studies evaluated cardiac rehabilitation attendance postoperatively.

Postoperative physical activity behavior

For peer review only

The impact of preoperative physical activity on postoperative physical activity levels was examined in three studies (Table 3).[12,14,21] These studies found that the active group preoperatively was more likely to be physically inactive postoperatively. Two of three studies completed a multivariate analyses and this association remained statistically significant after controlling for age, gender, and preoperative clinical characteristics.[12,14]

Risk of bias

The risk of bias assessment via the Newcastle-Ottawa Scale can be viewed in the Supplemental Digital Content. Since some studies assessed multiple outcomes, the risk of bias assessments were based on their highest possible score (e.g., some outcomes were assessed with a multivariable analysis, while others were not in the same study). All studies scored at least 3 out of 4 for the selection of study groups. There was variability across studies for the ascertainment of exposure or outcome of interest. Total risk of bias scores ranged from 5 to 9, suggesting the studies were of moderate to high quality, respectively.

For peer review only

DISCUSSION

The purpose of this systematic review was to determine if physical activity before cardiac surgery was associated with postoperative health outcomes. Given the different self-reported physical activity tools used, the inconsistent use of adjustment for potential confounders, and the varying outcomes evaluated for MACCE and 30 day postoperative events, it cannot be concluded that preoperative physical activity is associated with postoperative outcomes in adult cardiac surgery patients. This systematic review highlight important gaps within the literature on this topic. Therefore, key recommendations for examining the impact of preoperative physical activity behavior on post-surgical outcomes of cardiac patients are provided (Box 1).

The different self-reported physical activity tools used across the studies makes it difficult to compare the preoperative physical activity levels of patients prior to cardiac surgery. There seems to be no universally accepted tool to measure self-reported physical activity levels.[23] One advantage of using self-reported physical activity measures in studies is their ease of administration compared to other objectively measured physical activity tools. Furthermore, self-reported physical activity tools appear to provide some value when assessing the independent association between activity levels and poor outcomes. In fact, most physical activity guideline recommendations for health benefits, including those in North America, are based on self-reported measures.[24,25] However, cardiac surgery patients and other patient populations tend to misreport their physical activity levels compared to objectively measured physical activity (e.g., accelerometers).[6,26] Nevertheless, this systematic review found no studies that evaluated objectively measured physical activity before cardiac surgery and its link to postoperative health outcomes. Evidence suggests there is a stronger association between objective measures of physical activity and various cardiovascular and metabolic biomarkers as compared to subjective measures of physical activity.[27,28] Therefore, future studies should use a physical activity tools such as accelerometers or pedometers.

For peer review only

There were inconsistent findings across studies assessing the same outcomes, and many studies did not adjust for clinically relevant variables that could influence the health outcomes of cardiac surgery patients. Even so, some of the results of this systematic review are promising. Specifically, of the studies which controlled for confounding variables, five studies found a protective, independent association, between higher preoperative physical activity levels when assessing clinical outcomes, including MACCE,[17] 30 day postoperative events,[16,18] hospital length of stay,[16] and ICU length of stay;[19] whereas, only two studies found no protective association for 30 day postoperative events[20] and postoperative ADLs.[19] Yet, more studies are needed to elucidate the impact of preoperative physical activity on post-cardiac surgical outcomes that control for clinically relevant variables. Clinical variables included in the cardiac surgical risk models (e.g., EuroSCORE, STS score) could attenuate or mitigate the relationship between preoperative physical activity behavior and postoperative outcomes. Collectively, future studies are needed to determine if preoperative physical activity is a protective factor for health outcomes after cardiac surgery which control for clinically relevant variables known to impact cardiac surgery outcomes.

An unanticipated finding was that patients who were active before surgery had a higher likelihood of being physically inactive postoperatively, after controlling for co-morbidities.[12,14,21] Healthcare providers may have advised patients with more severe symptomology prior to surgery to refrain from physical activity. Also, the relief of cardiac symptoms after surgery among inactive patients could have led them to become more active postoperatively. However, these possibilities were not explored in the included studies.

While outside the scope of this systematic review, future studies should investigate if changes to physical activity levels prior to cardiac surgery impact long-term patient health-outcomes. Cardiac rehabilitation programs are intended to support cardiac patients in becoming more physically active postoperatively and it has been shown that patients who attend such programs reduce their risk for cardiac-related mortality and hospitalization rates.[29] Evidence suggests that among those referred to

For peer review only

cardiac rehabilitation after cardiac surgery, only 40% attend.[6] However, the literature is less clear on whether patients who attend cardiac rehabilitation are more physically active compared to those who do not attend. It is possible that patients who adopt and sustain a more physically active lifestyle on their own after cardiac surgery could yield similar health benefits compared to those who attend an exercise-based rehabilitation program, but this hypothesis requires further investigation.

Previous randomized controlled trials comparing an exercise program to standard care prior to elective cardiac surgery (i.e., "Prehab") demonstrate reductions in hospital length of stay and improvements in walking ability postoperatively.[7–9] However, there were mixed findings from this systematic review when comparing preoperative physical activity behavior and hospital stay.[12,16] These divergent findings suggest either that a medically supervised and individualized physical activity program is needed to derive the health benefits of physical activity prior to cardiac surgery, or that patients are misreporting their physical activity behaviors. Future cohort studies in this area should address the drawbacks of the included studies in this systematic review included in Box 1, while randomized trials should focus on whether preoperative exercise therapy programs are feasible and efficacious in clinical practice.

The findings of this systematic review suggest that the literature would benefit from standardization of the definition of measures such as MACCE and postoperative events within 30 days. The heterogeneity in reporting of outcomes can lead to considerably different conclusions across studies.[30] Attempts should also be made to ensure other clinically important outcomes are captured, such as the addition of 30-day events. Only one study in this review compared physically active versus inactive patients preoperatively and reported on the individual postoperative events within 30 days.[20] Collectively, uniform outcome reporting and appropriate outcome definitions are recommended when examining the outcomes of cardiac surgery.[30]

For peer review only

Patient-oriented outcomes should also be captured to ensure that cardiac surgery is improving other outcomes that patients value. No studies in this review determined if there was a link between preoperative physical activity behavior and postoperative health-related QOL, and only one study evaluated postoperative ADLs.[19] QOL postoperatively tends to improve in some older patients, while others tend to decline.[31] Importantly, the preoperatively physical activity and overall functional status of cardiac surgery patients could play a role in the postoperative trajectory of these outcomes such as QOL. Other patient-oriented outcomes, including postoperative pain and cardiac symptoms, could also be investigated.

Limitations

One limitation to consider is that the patients included across the studies evaluated in this systematic review may have been different, as the recruitment criteria were not always clearly stated. A small sample of studies explicitly stated that they excluded those with physical limitations and healthcare providers may have advised higher risk patients to not participate in physical activity. There is also a limitation associated with the methodology of this systematic review: only studies written in English were included, raising the possibility that some studies were missed.

Conclusion

Due to the mixed findings in this systematic review, it cannot be concluded that self-reported physical activity behavior before cardiac surgery is associated with health outcomes after surgery. The mixed findings could be due to the heterogeneity in physical activity tools used, definitions of outcomes, and the few studies adjusting for other potentially confounding variables. These findings highlight the need for more research in this area.

For peer review only

385 **REFERENCES**

386 1. Ferguson TB, Hammill BG, Peterson ED, DeLong ER, Grover FL, STS National Database
387 Committee. A decade of change--risk profiles and outcomes for isolated coronary artery bypass
388 grafting procedures, 1990-1999: a report from the STS National Database Committee and the Duke
389 Clinical Research Institute. Society of Thoracic Surgeons. *Ann Thorac Surg*. 2002;73(2):480-489-
390 490.

391 2. Pfisterer M, Buser P, Osswald S, et al. Outcome of elderly patients with chronic symptomatic
392 coronary artery disease with an invasive vs optimized medical treatment strategy: one-year results
393 of the randomized TIME trial. *JAMA*. 2003;289(9):1117-1123.

394 3. Alexander KP, Anstrom KJ, Muhlbaier LH, et al. Outcomes of cardiac surgery in patients > or = 80
395 years: results from the National Cardiovascular Network. *J Am Coll Cardiol*. 2000;35(3):731-738.

396 4. Anderson L, Oldridge N, Thompson DR, et al. Exercise-Based Cardiac Rehabilitation for Coronary
397 Heart Disease: Cochrane Systematic Review and Meta-Analysis. *J Am Coll Cardiol*. 2016;67(1):1-
398 12. doi:10.1016/j.jacc.2015.10.044.

399 5. Macchi C, Polcaro P, Cecchi F, et al. One-year adherence to exercise in elderly patients receiving
400 postacute inpatient rehabilitation after cardiac surgery. *Am J Phys Med Rehabil Assoc Acad*
401 *Physiatr*. 2009;88(9):727-734.

402 6. Horne D, Kehler DS, Kaoukis G, et al. Impact of Physical Activity on Depression After
403 CardiacSurgery. *Can J Cardiol*. 2013;29(12):1649-1656.

404 7. Arthur HM, Daniels C, McKelvie R, Hirsh J, Rush B. Effect of a preoperative intervention on
405 preoperative and postoperative outcomes in low-risk patients awaiting elective coronary artery
406 bypass graft surgery. A randomized, controlled trial. *Ann Intern Med*. 2000;133(4):253-262.

407 8. Sawatzky JV, Kehler DS, Ready AE, et al. Prehabilitation program for elective coronary artery
408 bypass graft surgery patients: a pilot randomized controlled study. *Clin Rehabil*. 2013;In press.

409 9. Herdy AH, Marcchi PL, Vila A, et al. Pre- and postoperative cardiopulmonary rehabilitation in
410 hospitalized patients undergoing coronary artery bypass surgery: a randomized controlled trial. *Am J*

For peer review only

Phys Med Rehabil Assoc Acad Physiatr. 2008;87(9):714-719. doi:10.1097/PHM.0b013e3181839152.

10. Society of Thoracic Surgeons. General Thoracic Surgery Database. http://www.sts.org/sts-national-database/database-managers/general-thoracic-surgery-database. Accessed February 17, 2015.
11. Wells GA, Shea B, O'Connell D, et al. The Newcastle-Ottawa Scale (NOS) for assessing the quality of nonrandomised studies in meta-analyses. http://www.ohri.ca/programs/clinical_epidemiology/oxford.asp. Published 2011. Accessed January 25, 2015.
12. Markou AL, Lasten PJ, Noyez L. Physical activity post myocardial revascularization. Will surgery improve my mobility?. *J Cardiovasc Surg (Torino)*. 2007;48(2):201-206.
13. Nery RM, Barbisan JN, Mahmud MI. Influence of the practice physical activity in the coronary artery bypass graft surgery results. *Braz J Cardiovasc Surg*. 2007;22(3):297-302.
14. Markou AL, Evers M, van Swieten HA, Noyez L. Gender and physical activity one year after myocardial revascularization for stable angina. *Interact Cardiovasc Thorac Surg*. 2008;7(1):96-100.
15. Martini MR, Barbisan JN. Influence of physical activity during leisure time in patients in the follow-up two years after CABG. *Rev Bras Cir Cardiovasc Orgao Of Soc Bras Cir Cardiovasc*. 2010;25(3):359-364.
16. Nery RM, Barbisan JN. Effect of leisure-time physical activity on the prognosis of coronary artery bypass graft surgery. *Rev Bras Cir Cardiovasc Orgao Of Soc Bras Cir Cardiovasc*. 2010;25(1):73-78.
17. Rengo G, Galasso G, Vitale DF, et al. An active lifestyle prior to coronary surgery is associated with improved survival in elderly patients. *J Gerontol A Biol Sci Med Sci*. 2010;65(7):758-763. doi:10.1093/gerona/glp216.
18. Giaccardi M, MacChi C, Colella A, et al. Postacute rehabilitation after coronary surgery: The effect of preoperative physical activity on the incidence of paroxysmal atrial fibrillation. *Am J Phys Med Rehabil*. 2011;90(4):308-315.

For peer review only

For peer review only - http://bmjopen.bmj.com/site/about/guidelines.xhtml

437 19. Cacciatore F, Anello CB, Ferrara N, et al. Determinants of prolonged intensive care unit stay after cardiac surgery in the elderly. *Aging Clin Exp Res*. 2012;24(6):627-634.

439 20. Noyez L, Biemans I, Verkroost M, van Swieten H. Is a sedentary lifestyle an independent predictor for hospital and early mortality after elective cardiac surgery?. *Neth Heart J*. 2013;21(10):439-445.

441 21. Min L, Mazzurco L, Gure TR, et al. Longitudinal functional recovery after geriatric cardiac surgery. *J Surg Res*. 2015;194(1):25-33.

443 22. van Laar C, Kievit PC, Noyez L. Surgical aortic valve replacement in patients older than 75 years: is there really a quality of life benefit?. *Neth Heart J*. 2015;23(3):174-179.

445 23. van Poppel MN, Chinapaw MJ, Mokkink LB, van Mechelen W, Terwee CB. Physical activity questionnaires for adults: a systematic review of measurement properties. *Sports Med Auckl NZ*. 2010;40(7):565-600. doi:10.2165/11531930-000000000-00000;

448 24. Tremblay MS, Warburton DER, Janssen I, et al. New Canadian physical activity guidelines. *Appl Physiol Nutr Metab Physiol Appliquée Nutr Métabolisme*. 2011;36(1):36-46-58. doi:10.1139/H11-009.

451 25. Physical Activity Guidelines Advisory Committee. *Physical Activity Guidelines Advisory Committee Report.* Washington, DC: U.S.: Department of Health and Human Services; 2008.

453 26. Lee PH, Macfarlane DJ, Lam TH, Stewart SM. Validity of the International Physical Activity Questionnaire Short Form (IPAQ-SF): a systematic review. *Int J Behav Nutr Phys Act*. 2011;8. doi:10.1186/1479-5868-8-115.

456 27. Schmidt MD, Cleland VJ, Thomson RJ, Dwyer T, Venn AJ. A comparison of subjective and objective measures of physical activity and fitness in identifying associations with cardiometabolic risk factors. *Ann Epidemiol*. 2008;18(5):378-386. doi:10.1016/j.annepidem.2008.01.005.

459 28. Atienza AA, Moser RP, Perna F, et al. Self-reported and objectively measured activity related to biomarkers using NHANES. *Med Sci Sports Exerc*. 2011;43(5):815-821. doi:10.1249/MSS.0b013e3181fdfc32.

For peer review only

29. Anderson L, Thompson DR, Oldridge N, et al. Exercise-based cardiac rehabilitation for coronary heart disease. *Cochrane Database Syst Rev*. 2016;1:CD001800. doi:10.1002/14651858.CD001800.pub3.

30. Goldfarb M, Drudi L, Almohammadi M, et al. Outcome Reporting in Cardiac Surgery Trials: Systematic Review and Critical Appraisal. *J Am Heart Assoc*. 2015;4(8):e002204. doi:10.1161/JAHA.115.002204.

31. Abah U, Dunne M, Cook A, et al. Does quality of life improve in octogenarians following cardiac surgery? A systematic review. *BMJ Open*. 2015;5(4):e006904. doi:10.1136/bmjopen-2014-006904.

For peer review only

FIGURE LEGENDS

Figure 1. Study flow diagram.

For peer review only

Table 1. Characteristics of included studies.

First author, year	Study Population	Country	Participants at follow-up	Physical Activity Assessment	Longest follow-up	Main findings
Giaccardi, 2011[18]	All patients ≥ 65 years undergoing CABG and/or valve procedures (total sample: 74.1 ± 5.8 years old); 43% female	Italy	158	Harvard Alumni Questionnaire	Four weeks postoperatively	Physical activity had an independent association with postoperative atrial fibrillation within 30 days.
Markou, 2007[12]	Elective CABG patients (Active: 64.4 ± 9.4, Inactive: 63.8 ± 9.0 years old); % female not reported	Netherlands	428	The Corpus Christi Heart Project	One year	Inactive vs. Active group had significantly more peri-operative MIs, but not reoperations, ICU LOS, HLOS, or postoperative complications at one year. Inactive group was more likely than Active group to be physically active at one year.
Nery, 2007[13]	All patients undergoing CABG (Active: 63 ± 11, Inactive 66 ± 14 years old); 42% female	Brazil	55	Structured Questionnaire confirmed by Minnesota Leisure Time Physical Activity Questionnaire	One year	Inactive vs. Active group had significantly longer HLOS and more postoperative events at one year.
Markou, 2008[14]	Elective CABG patients (64.3 ± 9.04 years old); 18% female	Netherlands	568	The Corpus Christi Heart Project	One year	Inactive vs. Active group were more likely to be more physically active one year postoperatively.
Martini, 2010[15]	Elective CABG patients (Active: 60 ± 10, Inactive: 62 ± 10 years old); 34% female	Brazil	185	Baecke Usual Physical Activity Questionnaire	Two years	Inactive vs. Active group did not have significantly different MACCE outcomes at two years.

For peer review only

Nery, 2010[16]	Elective CABG patients (Active: 60 ± 10, Inactive: 62 ± 10 years old); 34% female	Brazil	202	Baecke Usual Physical Activity Questionnaire	Hospital discharge	Inactive vs. Active group had more postoperative events within 30 days and a longer HLOS.
Rengo, 2010[17]	Acute or elective CABG patients ≥ 70 years (Active: 72.3 ± 3.2, Inactive: 76.1 ± 3.9 years old); 34% female	Italy	587	Physical Activity Scale for the Elderly	Mean 44.3 ± 21.0 months	Physical activity had an independent and dose association with cardiac and all-cause mortality five years postoperatively.
Cacciatore, 2012[19]	All patients ≥ 65 years undergoing CABG and/or valve procedures (72.9 ± 4.8 years old); 48% female	Italy	250	Physical Activity Scale for the Elderly	Hospital discharge	Physical activity was independently associated with reduced prolonged ICU LOS. Physical activity was not independently associated with postoperative ADLs.
Noyez, 2013[20]	Elective CABG and/or valve patients (69.7 ± 10.1 years old);	Netherlands	3150	The Corpus Christi Heart Project	30 days postoperative ly	Physical activity was not independently associated with hospital or 30 day mortality. Inactive vs. Active group had a significantly longer ICU LOS.
Min, 2015[21]	Elective CABG and/or valve patients ≥ 65 years (74.7 ± 5.9 years old)	United States of America	62	The Health and Retirement Survey	4-6 months	Inactive vs. Active group had significantly higher postoperative physical activity up to 6 months postoperatively.
van Laar[22]	Patients ≥75 years undergoing elective isolated aortic valve replacement (79.5 ± 2.8 years old); 59% female	Netherlands	115	The Corpus Christi Heart Project	2 years postoperative ly	Inactive vs. Active group had significantly higher mortality rates 2 years postoperatively.

CABG, coronary artery bypass graft surgery; HLOS, hospital length of stay; ICU LOS, intensive care unit length of stay; MI, myocardial infarction; MACCE, major adverse cerebrovascular and cardiac events; ADL, activities of daily living.

For peer review only

Table 2. Major adverse and cerebrovascular events and postoperative events within 30 days

Reference	Outcome definition	Adjustment variables	Number of events per group	OR or HR and 95% CI
**Major adverse cerebrovascular and cardiac events**				
Nery, 2007[13]	One year postoperative AF, hospital readmission, new CABG, PCI, MI	None	Active: 8/25 (31%); Inactive: 17/30 (57%)[a]	NR
Martini, 2010[15]	Two year postoperative death, re-hospitalization, cerebrovascular accident, MI	None	Active: 9/66 (14%); Inactive: 31/119 (26%)	NR
Rengo, 2010[17]	Five-year postoperative cardiac and all-cause mortality	Demographics, medical history, medications, and clinical findings.	NR	Adjusted proportional hazard models:<br>*All-cause mortality:* Exp(B) 0.248 (95% CI 0.141-0.434)[a]<br>*Cardiac mortality:*<br>Exp(B) 0.272 (0.133-0.555)[a]
van Laar[22]	Two-year mortality	None	Active: 5/65 (13%); Inactive: 11/50 (22%)[a]	NR
**Postoperative events within 30 days**				
Markou, 2007[12]	Perioperative MI, Re-intervention, postoperative complications (wound, renal, neurological, pulmonary, gastrointestinal)	None	MI: Active: 4/226 (2%); Inactive: 11/202 (5%)[a]<br>Reoperation: Active: 15/226 (7%); Inactive: 9/202 (5%),<br>Wound infection: Active: 3/226 (1%); Inactive: 7/202 (3%),<br>Renal: Active: 3/226; Inactive: 7/202	NR
Nery, 2010[16]	Mortality, MI, reoperation	Age, smoking, PVD, COPD, Cleveland Risk Score.	Mortality: Active: 0/66 (0%); Inactive: 7/136 (5%)<br>MI: Active: 1/66 (2%); Inactive: 6/136 (4%)<br>Reoperation: Active: 0/66 (0%); Inactive: 1/136 (0.5%)	Multivariate OR for being active:<br>0.22 (95% CI 0.09-0.51, p=0.001)

For peer review only

Rengo, 2010[17]	Low-output syndromes, MI, cardiac support, stroke, bleedings, mediastinitis, pneumonia, dialysis	None	Any surgical complication: Active: 53/267 (19.7%); Inactive: 60/320 (18.6%)	NR
Giaccardi, 2011[18]	Atrial fibrillation	Age, episodes of AF one year preop, episodes of AF in the first week, β-blockers, amiodarone, left ventricular volume, left atrial emptying fraction	Postoperative atrial fibrillation: Active: 6/74 (8.1%); Inactive: 27/84 (32.1%)[a]	Multivariate OR for being inactive: 4.04 (95% CI 1.16-14.14, p=0.029)
Noyez, 2013[20]	Mortality, reoperation, stroke, renal insufficiency, sternal wound, ventilation	≥75 years, valve surgery, female, high operative risk, renal disease, obesity, NYHA IV, Insulin, vascular pathology, poor LVEF, lung disease, MI, neurological event	Hospital mortality: Active: 7/1815 (0.4%); Inactive: 15/1335 (1.1%)[a]<br>30 day mortality: Active: 10/1815 (0.6%); Inactive: 20/1335 (1.5%)[a]<br>Reoperation: Active: 105/1815 (5.8%); Inactive: 68/1335 (5%)<br>Stroke: Active: 9/1815 (0.5%); Inactive: 12/1335 (0.9%)<br>Renal insufficiency: Active: 32/1815 (1.8%); Inactive: 39/1335 (2.9%)[a]<br>Sternal wound: Active: 10/1815 (0.6%); Inactive: 17/1335 (1.3%)[a]<br>Ventilation >2 days: Active: 31/1815 (1.7%); Inactive: 54/1335 (4.0%)[a]	Hospital mortality multivariate OR for being inactive: 1.20 (95% CI 0.4-3.5, p=0.617)<br><br>30 day mortality multivariate OR for being inactive: 1.10 (95% CI 0.5-2.7, p=0.70)

[a] indicates statistical significance (P<0.05). CABG, coronary artery bypass graft; PCI, percutaneous coronary intervention; MI, myocardial infarction; NR, not reported; PVD, peripheral vascular disease; COPD, chronic obstructive pulmonary disease; OR, odds ratio; AF, atrial fibrillation; BMI, body mass index; NYHA, New York Heart Association; LVEF, left ventricular ejection fraction.

For peer review only

Table 3. Hospital length of stay, ICU length of stay, and postoperative activities of daily living and physical activity.

First author, year	Adjustment variables	Length of stay/number of events per group	Odds ratio (OR) or hazard ratio (HR) and 95% confidence interval (CI)
**Hospital length of stay**			
Markou, 2007[12]	None	Active: 6.9 ± 8.2 days; Inactive: 7.3 ± 7.1 days	NR
Nery, 2007[13]	None	Active: 12 ± 5 days, median 9 days (IQR 8-15); Inactive: 15 ± 8 days, median 12 (IQR 9-19)[a]	NR
Nery, 2010[16]	Age, sex, Cleveland Risk Score, smoking, systemic arterial hypertension, stroke, MI, and PVD.	NR	HR: 0.67 (95% CI 0.49-0.93)[a]
**ICU length of stay**			
Markou, 2007[12]	None	Active: 2.2 ± 5.3 days; Inactive: 2.1 ± 3.5 days	NR
Cacciatore, 2012[19]	For ICU LOS >3 days: age, off-pump CABG, stroke, renal failure.	Active: 2.58 ± 1.09 days; Inactive: 3.33 ± 1.68 days[a,b]	For ICU length of stay >3 days<br>Univariate OR: 0.984 (95% CI 0.977-0.992)[a]<br>Multivariate OR: 0.992 (95% CI 0.983-1.000)[a]
Noyez, 2013[20]	None	Active: 1.3 ± 1.9 days; Inactive 3.0 ± 41.8 days[a]<br><br>ICU > 5 days: Active: 19/1815 (1.0%); Inactive: 46/1335 (3.4%)[a]	NR
**Postoperative ADLs**			
Cacciatore,	Age, gender, CABG, NYHA ≥3,	NR	Beta: 0.099

For peer review only

2012[19]	ICU LOS ≥3 days, Off-pump CABG, diabetes, renal failure, stroke, PVD, COPD, Cumulative Illness Rating Scale.		
**Postoperative Physical activity**			
Markou, 2007[12]	Age ≥75 years, gender, neurological disease, vascular disease, diabetes, and preoperative physical activity.	Better PA post-operatively: Active: 48/226 (21.2 %), Inactive: 129/202 (64%) [a]<br><br>Equal PA post-operatively: Active: 112/226 (49.6%), Inactive: 59/202 (29.2%) [a]<br><br>Worse PA postoperatively: Active: 66/226 (29.2%), Inactive: 14/202 (6.9%) [a]	Decreased postoperative PA OR (inactive group as reference): 8.1 (95% CI 3.5-13.5) [a]
Markou, 2008[14]	Diabetes, vascular disease, neurological disease, renal disease, MI, preoperative activity level.	NR	For becoming physically inactive postoperatively<br>Male OR (inactive group as reference): 7.11 (95% CI 3.6-13.9) [a]<br><br>Female OR (inactive group as reference): 11.0 (95% CI 2.2-55) [a]
Min, 2015[21]	None	NR	Each weekly preoperative activity point was associated with a loss of 0.78 points at 6 weeks, p<0.001, and 0.65 points at 6 months) [a]

[a] indicates statistical significance (P<0.05). [b]Unpublished data obtained from Cacciatore et al, [19]. ICU, Intensive Care Unit; ADL; activities of daily living; IQR, interquartile range; NR, not reported; MI, myocardial infarction; PVD, peripheral vascular disease; HR, hazard ratio; OR, odds ratio; CABG, coronary artery bypass graft; NYHA, New York Heart Association; COPD, chronic obstructive pulmonary disease; PA, physical activity.

For peer review only

Table 4. Newcastle-Ottawa scale risk of bias scores

Reference	Selection	Comparability	Outcome	Total
Markou, 2007[12]	3	2	3	8
Nery, 2007[13]	3	0	2	5
Markou, 2008[14]	3	2	2	7
Martini, 2010[15]	3	0	2	5
Nery, 2010[16]	3	2	2	7
Rengo, 2010[17]	4	2	3	9
Giaccardi, 2011[18]	3	2	2	7
Cacciatore, 2012[19]	3	2	2	7
Noyez, 2013[20]	3	2	3	8
Min, 2015[21]	4	2	1	7
van Laar[22]	3	0	3	6
Average scores ± SD	3.18±0.40	1.45±0.93	2.27±0.65	6.91±1.22

Maximum scores are 4, 2, and 3 for selection, comparability, and outcome, respectively. Maximum total score is 9. A lower score within each category and for a total score indicates a higher risk of bias.

For peer review only

Box 1. Guidelines for physical activity measurement and outcome assessment in cardiac surgery patients: limitations and opportunities for future research

Drawbacks	Opportunity
**Physical activity**	
1. Heterogeneity in tools used across studies<br>2. Only subjective measures were used	-use of objectively measured tools (e.g., pedometers, accelerometers) which can produce data that can be compared across studies, such as step counts, intensity, and duration of physical activity.
3. Time of preoperative physical activity assessment was unclear in most studies	-Capture physical activity behavior as soon as a patient is placed on a wait list, or in non-elective cases, as soon as possible prior to surgery.<br>-Physical activity should be assessed ideally over a 7 day period.<br>-Physical activity should be assessed by intensity and duration per week, and in steps per day.
**Outcomes**	
4. Heterogeneity in MACCE and postoperative events within 30 days definitions	-MACCE should be evaluated as a long-term outcome and defined as death, stroke, myocardial infarction, and the need for re-do cardiac surgery. Each outcome should be evaluated individually.<br>-30-day postoperative events should be evaluated using the STS checklist:[10] along with reasons, evaluate unexpected return to the operating room, complications due to pulmonary, cardiovascular, gastrointestinal, hematological, urologic, infection, neurological, and other important miscellaneous outcomes (e.g., unexpected admission to ICU, or other events requiring admission to operating room requiring anesthesia.<br>-re-hospitalization for any cause after cardiac surgery should also be added to outcomes.
5. No patient-oriented outcomes were assessed	-Capture postoperative health-related quality of life, mental health, pain, and cardiac symptoms using validated tools within the first 30 days and at least one-year postoperatively.
**Statistical procedures**	
6. Shortage of studies addressing confounders	-use multivariate analysis, including logistic or linear regression, or analysis of variance statistical procedures. Ensure that a power analysis is conducted prior to conducting the study.

MACCE, major adverse cerebrovascular and cardiac events. STS, Society of Thoracic Surgeons. ICU, intensive care unit.

For peer review only

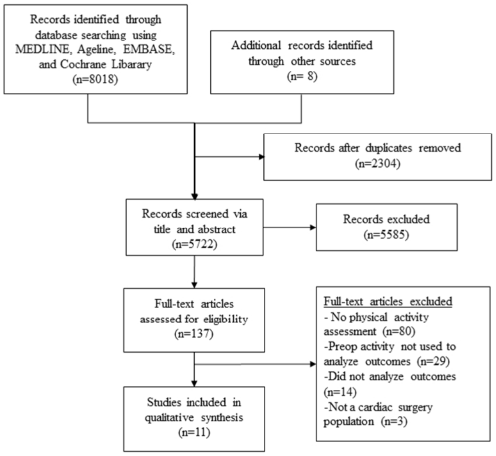

Figure 1. Study flow diagram

108x60mm (300 x 300 DPI)

review only

PRISMA 2009 Checklist

Section/topic	#	Checklist item	Reported on page #
**TITLE**			
Title	1	Identify the report as a systematic review, meta-analysis, or both.	1
**ABSTRACT**			
Structured summary	2	Provide a structured summary including, as applicable: background; objectives; data sources; study eligibility criteria, participants, and interventions; study appraisal and synthesis methods; results; limitations; conclusions and implications of key findings; systematic review registration number.	2
**INTRODUCTION**			
Rationale	3	Describe the rationale for the review in the context of what is already known.	3
Objectives	4	Provide an explicit statement of questions being addressed with reference to participants, interventions, comparisons, outcomes, and study design (PICOS).	3
**METHODS**			
Protocol and registration	5	Indicate if a review protocol exists, if and where it can be accessed (e.g., Web address), and, if available, provide registration information including registration number.	4
Eligibility criteria	6	Specify study characteristics (e.g., PICOS, length of follow-up) and report characteristics (e.g., years considered, language, publication status) used as criteria for eligibility, giving rationale.	4
Information sources	7	Describe all information sources (e.g., databases with dates of coverage, contact with study authors to identify additional studies) in the search and date last searched.	5
Search	8	Present full electronic search strategy for at least one database, including any limits used, such that it could be repeated.	5 (link provided)
Study selection	9	State the process for selecting studies (i.e., screening, eligibility, included in systematic review, and, if applicable, included in the meta-analysis).	5
Data collection process	10	Describe method of data extraction from reports (e.g., piloted forms, independently, in duplicate) and any processes for obtaining and confirming data from investigators.	5-6
Data items	11	List and define all variables for which data were sought (e.g., PICOS, funding sources) and any assumptions and simplifications made.	6
Risk of bias in individual studies	12	Describe methods used for assessing risk of bias of individual studies (including specification of whether this was done at the study or outcome level), and how this information is to be used in any data synthesis.	6
Summary measures	13	State the principal summary measures (e.g., risk ratio, difference in means).	No meta-analysis

For peer review only

PRISMA 2009 Checklist

Synthesis of results	14	Describe the methods of handling data and combining results of studies, if done, including measures of consistency (e.g., I^2) for each meta-analysis.	No meta-analysis

Section/topic	#	Checklist item	Reported on page #
Risk of bias across studies	15	Specify any assessment of risk of bias that may affect the cumulative evidence (e.g., publication bias, selective reporting within studies).	N/A
Additional analyses	16	Describe methods of additional analyses (e.g., sensitivity or subgroup analyses, meta-regression), if done, indicating which were pre-specified.	N/A
**RESULTS**			
Study selection	17	Give numbers of studies screened, assessed for eligibility, and included in the review, with reasons for exclusions at each stage, ideally with a flow diagram.	7
Study characteristics	18	For each study, present characteristics for which data were extracted (e.g., study size, PICOS, follow-up period) and provide the citations.	7 and in Table 1 (page 20-22)
Risk of bias within studies	19	Present data on risk of bias of each study and, if available, any outcome level assessment (see item 12).	10 and in Table 4 (page 28)
Results of individual studies	20	For all outcomes considered (benefits or harms), present, for each study: (a) simple summary data for each intervention group (b) effect estimates and confidence intervals, ideally with a forest plot.	8-10 and in tables 2 and 3 (pages 23-27)
Synthesis of results	21	Present results of each meta-analysis done, including confidence intervals and measures of consistency.	No meta-analysis
Risk of bias across studies	22	Present results of any assessment of risk of bias across studies (see Item 15).	10
Additional analysis	23	Give results of additional analyses, if done (e.g., sensitivity or subgroup analyses, meta-regression [see Item 16]).	N/A
**DISCUSSION**			
Summary of evidence	24	Summarize the main findings including the strength of evidence for each main outcome; consider their relevance to key groups (e.g., healthcare providers, users, and policy makers).	11

For peer review only

PRISMA 2009 Checklist

Limitations	25	Discuss limitations at study and outcome level (e.g., risk of bias), and at review-level (e.g., incomplete retrieval of identified research, reporting bias).	14
Conclusions	26	Provide a general interpretation of the results in the context of other evidence, and implications for future research.	14
**FUNDING**			
Funding	27	Describe sources of funding for the systematic review and other support (e.g., supply of data); role of funders for the systematic review.	None. Indicated in the title page

From: Moher D, Liberati A, Tetzlaff J, Altman DG, The PRISMA Group (2009). Preferred Reporting Items for Systematic Reviews and Meta-Analyses: The PRISMA Statement. PLoS Med 6(6): e1000097. doi:10.1371/journal.pmed1000097

For more information, visit: **www.prisma-statement.org**.

For peer review only

For peer review only - http://bmjopen.bmj.com/site/about/guidelines.xhtml

BMJ Open

Systematic Review of preoperative physical activity and its impact on post-cardiac surgical outcomes

Journal:	*BMJ Open*
Manuscript ID	bmjopen-2016-015712.R1
Article Type:	Research
Date Submitted by the Author:	31-May-2017
Complete List of Authors:	Kehler, D. Scott<br>Stammers, Andrew N.; University of Manitoba,<br>Tangri, Navdeep; University of Manitoba, Medicine<br>Hiebert, Brett<br>Fransoo, Randy; University of Manitoba, Community Health Sciences<br>Schultz, Annette; University of Manitoba, Nursing<br>Macdonald, Kerry<br>Giacomontonio, Nicholas<br>Hassan, Ansar; New Brunswick Heart Centre,<br>Légaré, Jean-Francois<br>Arora, Rakesh C.; University of Manitoba,<br>Duhamel, Todd A.; University of Manitoba,
**Primary Subject Heading**:	Cardiovascular medicine
Secondary Subject Heading:	Rehabilitation medicine
Keywords:	Cardiac surgery < SURGERY, REHABILITATION MEDICINE, Exercise, Postoperative complications, Prognosis

SCHOLARONE™
Manuscripts

For peer review only

Title: Systematic Review of preoperative physical activity and its impact on post-cardiac surgical outcomes

Authors

D. Scott Kehler, MSc,§[1,2] Andrew N. Stammers, B. Kin,[1,2] Navdeep Tangri, PhD,[3] Brett Hiebert, MSc,[4] Randy Fransoo, PhD,[5] Annette S. Schultz, RN, PhD,[6] Kerry Macdonald, MLIS,[7] Nicholas Giacomantonio, MD, FRCPC,[8] Ansar Hassan, MD, PhD, FRCSC,[9] Jean-Francois Légaré, MD, FRCSC,[10] Rakesh C. Arora, MD, PhD, FRCSC, FACS,†[4] and Todd A. Duhamel, PhD.†[1,2]

†Contributed equally as senior authors

§Corresponding author

Affiliations

[1]Health, Leisure & Human Performance Research Institute, Faculty of Kinesiology and Recreation Management, University of Manitoba, Winnipeg, Canada;

[2]Institute of Cardiovascular Sciences, St. Boniface Hospital Research Centre, Winnipeg, Canada;

[3]Seven Oaks Hospital Research Centre, Winnipeg, Canada;

[4]Department of Surgery, University of Manitoba and Cardiac Sciences Program, Winnipeg, Canada;

[5]Department of Community Health Sciences and Manitoba Centre for Health Policy, University of Manitoba, Winnipeg, Canada;

[6]College of Nursing, Faculty of Heath Sciences, University of Manitoba Winnipeg, Canada;

[7]Seven Oaks Hospital Library, Winnipeg, Canada;

[8]Division of Cardiology, Department of Medicine, Dalhousie University, Halifax, Canada;

[9]Department of Cardiac Surgery, New Brunswick Heart Centre, Saint John Regional Hospital, Saint John, Canada;

[10]Division of Cardiac Surgery, Department of Surgery, Dalhousie University, Halifax, Canada;

For peer review only

For peer review only - http://bmjopen.bmj.com/site/about/guidelines.xhtml

Address for correspondence:

1. Mr. Scott Kehler, 212 Active Living Centre, Winnipeg, MB, Canada, R3T 2N2

Telephone +1204 918 9131 Fax +1204 474 7634

e-mail: skehler@sbrc.ca

Acknowledgements:

Dr. Francesco Cacciatore and his research group provided additional data on ICU length of stay. TAD was supported by a Manitoba Health Research Council (MHRC) Establishment Grant. DSK was supported by an MHRC Studentship, A CIHR Frederick Banting and Charles Best Canada Graduate Scholarship, a CIHR Strategic Training Initiative in Health Research (STIHR) Knowledge Translation Canada Student Fellowship, a CIHR STIHR Population Intervention for Chronic Disease Prevention Fellowship and the Heart and Stroke Foundation Dr. Dexter Harvey Award. ANS was supported by a CIHR Canada Graduate Scholarship.

Authors' Contributions:

DSK was responsible for 1) analysis and interpretation of data, 2) drafting and revising the manuscript, and 3) consenting for manuscript submission. ANS, BH, NT, RF, ASH, NG, AH, JL were responsible for 1) analysis and interpretation of data, 2) revising the manuscript, and 3) consenting for manuscript submission. KM was responsible for 1) developing the systematic review literature search, 2) analysis and interpretation of data, 3) drafting and revising the manuscript, and 3) consenting for manuscript submission. RCA and TAD were responsible for 1) the conception and design, and analysis and interpretation of data, 2) revising the manuscript, and 3) consenting for manuscript submission RCA and TAD are co-senior authors.

Funding:

The work was supported by an Operating Grant from the Canadian Institutes of Health Research

For peer review only

grant number 326290.

Ethics Approval:

Not applicable

Systematic review registration number:

PROSPERO: CRD42015023606

Competing Interests:

The authors have no conflicts of interest to declare

Data Sharing Statement:

There are no additional unpublished data to share as a part of this systematic review.

Word count (not including abstract, tables, and references): 3990

Figures and tables: 5

For peer review only

ABSTRACT:

Objectives: The objective of this systematic review was to study the impact of preoperative physical activity levels on adult cardiac surgical patients' postoperative: 1) major adverse cardiac and cerebrovascular events (MACCE), 2) adverse events within 30 days, 3) hospital length of stay (HLOS), 4) intensive care unit length of stay (ICU LOS), 5) activities of daily living (ADLs), 6) quality of life, 7) cardiac rehabilitation attendance, and 8) physical activity behavior.

Methods: A systematic search of MEDLINE, Embase, AgeLine, and Cochrane library for cohort studies was conducted.

Results: Eleven studies (n=5,733 patients) met the inclusion criteria. Only self-reported physical activity tools were used. Few studies used multivariate analyses to compare active versus inactive patients prior to surgery. When comparing patients who were active versus inactive preoperatively, there were mixed findings for MACCE, 30 day adverse events, HLOS, and ICU LOS. Of the studies which adjusted for confounding variables, five studies found a protective, independent association between physical activity and MACCE (n= 1), 30 day postoperative events (n= 2), hospital length of stay (n= 1), and ICU length of stay (n= 1), but two studies found no protective association for 30 day postoperative events (n= 1) and postoperative ADLs (n= 1). No studies investigated if activity status before surgery impacted quality of life or cardiac rehabilitation attendance postoperatively. Three studies found that active patients prior to surgery were more likely to be inactive postoperatively.

Conclusion: Due to the mixed findings, the literature does not presently support that self-reported preoperative physical activity behavior is associated with postoperative cardiac-surgical outcomes. Future studies should objectively measure physical activity, clearly define outcomes, and adjust for clinically relevant variables.

Registration: PROSPERO number CRD42015023606.

Keywords: Cardiac Surgical Procedures, Exercise, Prognosis, Postoperative Complications

For peer review only

ARTICLE SUMMARY

Strengths and limitations of this study

- There were mixed findings regarding the impact of physical activity on post cardiac-surgical outcomes.
- Only self-reported physical activity tools were used.
- The multiple tools to measure physical activity and the variety of definitions of outcomes did not allow for a quantitative synthesis (meta-analysis).

For peer review only

INTRODUCTION

Recent reports suggest that more than half of cardiac surgeries are being performed on older adults who are more likely to be frail and have multiple co-morbidities.[1] While cardiac surgery has been shown to improve the outcomes of these patients, more than 75% of major perioperative complications and deaths occur in older adults.[2,3] Before surgery, many of these patients are de-conditioned and have diminished resilience in the face of major stressors such as cardiac surgery, and it has been postulated that they could benefit from a therapeutic intervention prior to their major surgical procedure in order to reduce their operative risk. However, little information exists to evaluate the benefit of preoperative risk reduction strategies for the older cardiac surgery patient.

Adopting and sustaining a more physically active lifestyle is typically intended to be a part of an interdisciplinary rehabilitation plan that is instituted postoperatively and has been shown to reduce the risk of cardiac mortality and hospital admissions and improve health-related (QOL) in patients.[4] Importantly, older adults who sustain a physically active lifestyle after a postoperatively exercise-based rehabilitation program can continue to improve their functional walking status.[5] However, evidence suggests that cardiac surgery patients are highly sedentary during the preoperative period, especially in older adults.[6] Furthermore, few randomized controlled trials exist which evaluate the therapeutic benefit of preoperative lifestyle modification in patients undergoing cardiac surgery.[7–9] Information regarding the link between preoperative physical activity and postoperative health outcomes in cardiac surgery patients would be valuable for healthcare providers to assist them in selecting patients who might benefit from preoperative exercise therapy.

The purpose of this systematic review was to compare the following postoperative outcomes between cardiac surgery patients defined as physically active prior to surgery and those who were defined as physically inactive preoperatively: 1) major adverse cerebrovascular and cardiovascular events (MACCE) 2) 30-day adverse events as defined by the Society of Thoracic Surgeons (STS)[10] 3) hospital length of

For peer review only

130 stay, 4) Intensive Care Unit (ICU) length of stay, 5) health-related QOL, 6) activities of daily living

131 (ADL) 7) cardiac rehabilitation attendance and 8) physical activity levels postoperatively.

132

For peer review only

MATERIAL AND METHODS

The protocol for this systematic review has been described in PROSPERO: CRD42015023606. Note the following *ad-hoc* changes to the previous protocol: ICU length of stay and postoperative physical activity as additional outcomes were explored in this systematic review.

Eligibility criteria

Eligible studies included cohort studies which examined adult (>18 years) cardiac surgery patients undergoing coronary artery bypass grafting (CABG), aortic or mitral valve repair/replacement, transcatheter aortic valve implantation, or combined procedures. Studies with patients undergoing congenital cardiac surgery, heart transplantation or left ventricular assist device implantation procedures were excluded. Studies could compare physically active versus inactive patients prior to cardiac surgery on the basis of subjective (e.g., questionnaire) or objective (e.g., pedometer, accelerometry) assessments of physical activity.

Eligible studies had to compare at least one of the following postoperative outcomes: MACCE defined as death, stroke, myocardial infarction, and the need for emergency cardiac surgery; 30-day adverse events as defined by the STS,[10] including an unexpected return to the operating room, complications due to pulmonary, cardiovascular, gastrointestinal, hematological, urologic, infection, and neurological deficits, other important miscellaneous outcomes (e.g., unexpected admission to ICU, or other events requiring admission to operating room requiring anesthesia; hospital length of stay; ICU length of stay; health-related QOL with any assessment tool; ADLs using any evaluation strategy; cardiac rehabilitation attendance; and physical activity behavior using either subjective or objective forms of assessment.

For peer review only

Search strategy

The search strategy was completed by a librarian and reviewed by a second librarian. The search included keywords and controlled vocabulary. English language limits were applied. Databases used included MEDLINE, Embase, AgeLine, and Cochrane Library (CDSR, CENTRAL, DARE) and articles were searched from inception to December 2016. The MEDLINE strategy was registered and published online in PROSPERO (http://www.crd.york.ac.uk/PROSPEROFILES/23606_STRATEGY_20150518.pdf) and is also available as a supplementary file. The search was validated through a cross-check of references of studies selected for inclusion. In addition, conference abstracts were hand searched using the Internet. Attempts were made to contact authors of conference abstracts to determine if their findings were published in a peer-reviewed journal.

Study selection

The title, abstract and full-text article screening processes were independently completed by two reviewers. A training exercise for the title and abstract phase was conducted by the independent reviewers using a random sample of 100 titles and abstracts. Discrepancies in studies for inclusion were resolved by discussion of the two reviewers. The final observed agreement was 98% with a kappa statistic of 0.47 for the title and abstract screen. One training exercise of 10 randomly selected articles was completed for the full-text screen. Discrepancies for inclusion were resolved through discussion. The observed agreement for the full-text screen was 96% with a kappa statistic of 0.83.

For peer review only

Data abstraction

Two reviewers independently extracted relevant data for the selected outcomes described above. Discrepancies in the data extraction procedure were resolved through discussion. Data abstraction items included study characteristics (e.g., authors, year of publication, sample size, follow up time points if relevant), patient characteristics (e.g., age, sex, surgery type), physical activity tool used, and the outcomes which were measured.

Risk of bias assessment

Two reviewers independently reviewed the risk of bias of each included study using the Newcastle-Ottawa Scale.[11] Items within this tool assess the risk of bias associated with selection of participants, comparability (e.g., study authors controlled for patient demographics and clinical characteristics), and outcome assessment (e.g., data collection method for outcome, sufficient follow-up, and adequacy of follow up of cohorts). Each study was given a score within each category (Selection: 0-4; Comparability: 0-2; and Outcome: 0-3) and an overall score ranging from 0-9. A score of zero suggests an increased risk of bias and a higher score suggests a lower risk of bias.

Quantitative synthesis

Due to the significant heterogeneity between studies in terms of physical activity assessment tools used and outcomes assessed, meta-analyses were not performed.

For peer review only

RESULTS

The literature search results are shown in Figure 1. After removing duplicates, 5722 articles were title and abstract screened. A total of 137 articles were then assessed in full-text. Eleven studies met the eligibility criteria for the final analysis, and they included a total of 5,733 patients.[12–22]

An overview of the included studies can be viewed in Table 1. In the studies by Markou et al.,[12,14] Nery et al.,[13,16] Martini et al.,[15] they evaluated CABG only patients. Rengo et al.,[17] Giaccardi et al.,[18] Cacciatore et al.,[19] Noyez et al.,[20] and Min et al.[21] evaluated both CABG and/or valve procedures, and van Laar et al.[22] evaluated isolated aortic valve repair patients. The average age of participants in different studies ranged from 60 years (Martini and Nery et al.[15,16]) to 75 years (Rengo, Giaccardi, Min, and van Laar et al[17,18,21,22]). Rengo et al.,[17] Giaccardi et al.,[18] Min et al.,[21] and van Laar et al.[22] excluded patients with physical impairments or with New York Heart Association heart failure class IV symptoms (severe cardiac symptoms) but in general exclusion criteria were not explicitly reported. Studies were conducted in the Netherlands (Markou et al.,[12,14] Noyez et al.,[20] and van Laar et al.[22]), Brazil (Nery et al.,[13,16] and Martini et al.[e]), Italy (Rengo et al.,[17] Giaccardi et al.,[18] and Cacciatore et al.[18]), and the United States (Min et al.[21]). Two studies by Nery et al.[13] and Martini et al.[15] used the same patient sample, but examined different outcomes. The sample size of studies ranged from 35 in the Min et al.[21] study to 3150 in the Noyez et al.[20] study

Physical activity tools

The physical activity assessments in each study were based on self-reported assessment tools. The timing of the physical activity assessments prior to surgery was not reported by Cacciatore et al.,[19] Nery et al.,[13,16] Markou et al.,[12,14] or by Martini et al.[15] Rengo et al.[17] reported the timing of their physical activity assessment, which was within 35±6 days prior to surgery. Noyez et al., and van Laar et al. measured

For peer review only

activity the day before surgery.[20,22] Min and colleagues measured physical activity four weeks prior to the patients' surgical procedure.[21] Finally, Giaccardi et al. measured preoperative physical activity levels approximately one week following surgery.[18]

Four studies used the Corpus Christi Heart Project questionnaire[12,14,20,22] which asks participants about their typical physical activity behaviors over the past year during their leisure time. Participants were categorized into a sedentary group if they accumulated less than 30 minutes per day of light intensity activity, or into an active group if they accumulated at least one session per week of dynamic activity lasting ≥15 minutes marked by moderate intensity. Nery et al.,[13,16] and Martini et al.[15] used a structured questionnaire confirmed by the Minnesota Leisure Time Physical Activity Questionnaire[13] or the Baecke Usual Physical Activity questionnaire.[15,16] Both physical activity tools ask participants to recall their usual activities 12 months prior and determine the frequency, intensity, and time of activity. Participants were categorized into an inactive group if they engaged only in light intensity (<3 metabolic equivalents) activity or into an active group if they achieved ≥3 metabolic equivalents. Rengo et al.[17] and Cacciatore et al.[19] used the Physical Activity Scale for the Elderly, which is a 7-day recall of a participants' frequency, intensity, duration, and type of activity. Participants receive a total score from 0-400. Rengo et al.[17] separated participants by inactive and active groups using the median score, whereas Cacciatore et al.[19] used the continuous measure. The Harvard Alumni Questionnaire was implemented by Giaccardi and colleagues[18] which measures the typical weekly amount and intensity of physical activity over the past year. Participants were categorized as inactive if they participated in <1 hour per week of light activity and as active if they participated in either ≥4 hours of light or more than 1-2 hours of moderate activity per week. In the study by Min et al.,[21] the physical activity-related questions were used from the Health and Retirement Survey, which determines a participants' frequency and intensity of activity in a typical week. These authors used the continuous score in their study.

MACCE

For peer review only

Outcomes within the definition of MACCE were evaluated in four studies (Table 2) by Nery et al.,[13] Martini et al.,[15] Rengo et al.,[17] and van Laar et al.[22] The follow-up periods were one (Nery et al.[13]) two (Martini et al.,[15] and van Laar et al.[22]) and five years (Rengo et al.[17]) postoperatively. Unadjusted differences between active versus inactive patients and MACCE (defined as atrial fibrillation, hospital admission, reoperation and MI) were found one-year postoperatively in the Nery et al.[13] study. The Martini et al.[15] study found no differences (defined as mortality, re-hospitalization, cerebrovascular accident and MI) at two years postoperatively The unadjusted rates of mortality within two years post-surgery was significantly higher in the active versus inactive group were found in the study by van Laar and colleagues[22] The study by Rengo and associates found a significant and dose-response relationship between physical activity and postoperative cardiac and all-cause mortality after controlling for preoperative demographics, medical history, medications, and clinical characteristics.[17]

30-day events

Five studies (Markou et al.,[12] Nery et al.,[16] Rengo et al.,[17] Giaccardi et al.,[18] and Noyez et al.[20]) evaluated postoperative events within 30 days of surgery (Table 2). The postoperative events measured varied significantly between the studies. Three studies (Nery et al.,[16] Giaccardi et al.,[18] and Noyez et al.[20]) examined if physical activity was an independent protective factor against postoperative events. Physical activity was an independent protective factor against the combined outcome of mortality, MI, and reoperation in the study by Nery et al.;[16] as well as postoperative atrial fibrillation in the Giaccardi and associates study;[18] but was not significant for in-hospital or 30-day mortality in the Noyez et al.[20] study.

Postoperative health-related QOL

No studies evaluated postoperative health-related QOL.

For peer review only

Hospital and ICU length of stay

Three studies by Markou et al.[12] and Nery et al.[13,16] compared hospital length of stay between active vs. inactive cardiac surgery patients (Table 3). Hospital length of stay was longer in the inactive group in two of three studies (both by Nery et al.[13,16]). One of the studies by Nery et al.[16] did not report hospital length of stay summary statistics between the active vs. inactive groups. However that study reported an independent association between the preoperative active vs. inactive group and a reduced likelihood of prolonged hospital length of stay, though "prolonged" was not defined in the study.

Three studies compared ICU length of stay between the preoperative physical activity groups (Table 3) (Markou et al.,[12] Cacciatore et al.,[19] and Noyez et al.[20]). Two studies (Markou et al.[12] and Noyez et al.[20]) found that the inactive group had a significantly longer ICU length of stay compared to the active group. In the study by Cacciatore and colleagues, they found in their multivariate analysis that the active group was less likely to have a prolonged ICU length of stay >3 days compared to the inactive group after controlling for age, off-pump CABG, stroke, and renal failure.

Postoperative ADLs

One study by Min et al.[19] examined the impact of preoperative physical activity and postoperative ADLs at the time of hospital discharge and revealed no statistically significant ($p= 0.079$) association between the two after adjusting for preoperative demographics and clinical variables.

Cardiac rehabilitation attendance

For peer review only

No studies evaluated cardiac rehabilitation attendance postoperatively.

Postoperative physical activity behavior

The impact of preoperative physical activity on postoperative physical activity levels was examined in the two studies by Markou et al.[12,14] and in the other study by Min et al.[21](Table 3).These studies found that the active group preoperatively was more likely to be physically inactive postoperatively. In both of the Markou et al. [12,14] studies, they completed a multivariate analyses and found that this association remained statistically significant after controlling for age, gender, and preoperative clinical characteristics.

Risk of bias

The risk of bias assessment via the Newcastle-Ottawa Scale can be viewed in Table 4. Since some studies assessed multiple outcomes, the risk of bias assessments were based on their highest possible score (e.g., some outcomes were assessed with a multivariable analysis, while others were not in the same study). All studies scored at least 3 out of 4 for the selection of study groups. There was variability across studies for the ascertainment of exposure or outcome of interest. Total risk of bias scores ranged from 5 to 9, suggesting the studies were of moderate to high quality, respectively.

For peer review only

DISCUSSION

The purpose of this systematic review was to determine if physical activity before cardiac surgery was associated with postoperative health outcomes. Given the different self-reported physical activity tools used that prevented comparison across studies, the inconsistent use of adjustment for potential confounders, and the varying outcomes evaluated for MACCE and 30 day postoperative events, it cannot be concluded that preoperative physical activity is associated with postoperative outcomes in adult cardiac surgery patients. This systematic review highlights important gaps within the literature on this topic. Therefore, key recommendations for examining the impact of preoperative physical activity behavior on post-surgical outcomes of cardiac patients are provided (Box 1).

The different self-reported physical activity tools used across the studies makes it difficult to compare the preoperative physical activity levels of patients prior to cardiac surgery. Even so, it is important to note that in the studies included in this systematic review, most of the studies identified a sub-sample of cardiac surgery patients who were more vulnerable to poor health outcomes by categorizing patients as active or inactive prior to surgery using their self-reported physical activity measures. However, the way the physical activity tools measured physical activity (e.g., over the past year or in the past week; see the Methods section) could have influenced the outcomes of the study. There seems to be no universally accepted tool to measure self-reported physical activity levels,[23] and it is unclear if any of the physical activity tools identified by this review have been validated in the cardiac surgery patient. One advantage of using self-reported physical activity measures in studies is their ease of administration compared to other objectively measured physical activity tools. Furthermore, self-reported physical activity tools appear to provide some value when assessing the independent association between activity levels and poor outcomes. In fact, most physical activity guideline recommendations for health benefits, including those in North America, are based on self-reported measures.[24,25] Another strength of using a subjective physical activity tool in the preoperative cardiac surgery patient is that it would capture a patient's physical activity behavior before they are placed on a waiting list, when they might refrain from being

For peer review only

physically active in fear of making their condition worse. However, cardiac surgery patients and other patient populations tend to misreport their physical activity levels compared to objectively measured physical activity.[6,26] Nevertheless, this systematic review found no studies that evaluated objectively measured physical activity before cardiac surgery and its link to postoperative health outcomes. Evidence suggests there is a stronger association between objective measures of physical activity and various cardiovascular and metabolic biomarkers as compared to subjective measures of physical activity.[27,28] While it is unclear which objective measures of physical activity are most appropriate in the complex cardiac surgical patients, future studies should use a physical activity tools such as accelerometers or pedometers.

There were inconsistent findings across studies assessing the same outcomes, and many studies did not adjust for clinically relevant variables that could influence the health outcomes of cardiac surgery patients. It is possible that most of the included studies were not statistically powered to detect changes between inactive and active groups. The study by Rengo et al.[17] had the largest sample size of the four studies that assessed MACCE outcomes, which found a significant protective association between preoperative physical activity and cardiac and all-cause mortality five years postoperatively after controlling for clinically relevant variables (Table 2). In contrast, the largest study examined in this systematic review by Noyez and colleagues[20] found no association between preoperative activity and hospital and 30-day mortality after controlling for covariates (Table 2). It is difficult to determine if patient-level factors influence outcomes (e.g., elective or acute patients, surgery type, older versus younger, females vs. males) as the samples were somewhat heterogeneous. Even so, some of the results of this systematic review are promising. Specifically, of the studies which controlled for confounding variables, five studies found a protective, independent association, between higher preoperative physical activity levels when assessing clinical outcomes, including MACCE,[17] 30 day postoperative events,[16,18] hospital length of stay,[16] and ICU length of stay;[19] whereas, only two studies found no protective association for 30 day postoperative events[20] and postoperative ADLs.[19] Yet, more studies are needed to

For peer review only

elucidate the impact of preoperative physical activity on post-cardiac surgical outcomes that control for clinically relevant variables. Clinical variables included in the cardiac surgical risk models (e.g., EuroSCORE, STS score) could attenuate or mitigate the relationship between preoperative physical activity behavior and postoperative outcomes. Collectively, future studies are needed to determine if preoperative physical activity is a protective factor for health outcomes after cardiac surgery which control for clinically relevant variables known to impact cardiac surgery outcomes.

An unanticipated finding was that patients who were active before surgery had a higher likelihood of being physically inactive postoperatively, after controlling for co-morbidities.[12,14,21] Healthcare providers may have advised patients with more severe symptomology prior to surgery to refrain from physical activity. Also, the relief of cardiac symptoms after surgery among inactive patients could have led them to become more active postoperatively. However, these possibilities were not explored in the included studies.

While outside the scope of this systematic review, future studies should investigate if changes to physical activity levels prior to cardiac surgery impact long-term patient health-outcomes. Cardiac rehabilitation programs are intended to support cardiac patients in becoming more physically active postoperatively and it has been shown that patients who attend such programs reduce their risk for cardiac-related mortality and hospitalization rates.[29] Evidence suggests that among those referred to cardiac rehabilitation after cardiac surgery, only 40% attend.[6] However, the literature is less clear on whether patients who attend cardiac rehabilitation are more physically active compared to those who do not attend. It is possible that patients who adopt and sustain a more physically active lifestyle on their own after cardiac surgery could yield similar health benefits compared to those who attend an exercise-based rehabilitation program, but this hypothesis requires further investigation.

For peer review only

Previous randomized controlled trials comparing an exercise program to standard care prior to elective cardiac surgery (i.e., "Prehab") demonstrate reductions in hospital length of stay and improvements in walking ability postoperatively.[7–9] However, there were mixed findings from this systematic review when comparing preoperative physical activity behavior and hospital stay.[12,16] These divergent findings suggest either that a medically supervised and individualized physical activity program is needed to derive the health benefits of physical activity prior to cardiac surgery, or that patients are misreporting their physical activity behaviors. Future cohort studies in this area should address the drawbacks of the included studies in this systematic review included in Box 1, while randomized trials should focus on whether preoperative exercise therapy programs are feasible and efficacious in clinical practice.

The findings of this systematic review suggest that the literature would benefit from standardization of the definition of measures such as MACCE and postoperative events within 30 days. The heterogeneity in reporting of outcomes can lead to considerably different conclusions across studies.[30] Attempts should also be made to ensure other clinically important outcomes are captured, such as the addition of 30-day events. Only one study in this review compared physically active versus inactive patients preoperatively and reported on the individual postoperative events within 30 days.[20] Collectively, uniform outcome reporting and appropriate outcome definitions are recommended when examining the outcomes of cardiac surgery.[30]

Patient-oriented outcomes should also be captured to ensure that cardiac surgery is improving other outcomes that patients value. No studies in this review determined if there was a link between preoperative physical activity behavior and postoperative health-related QOL, and only one study evaluated postoperative ADLs.[19] QOL postoperatively tends to improve in some older patients, while others tend to decline.[31] Importantly, the preoperatively physical activity and overall functional status of cardiac surgery patients could play a role in the postoperative trajectory of these outcomes such as QOL.

For peer review only

Other patient-oriented outcomes, including postoperative pain and cardiac symptoms, could also be investigated.

If physical activity is to be assessed in the preoperative period, the extent of missing data may also be a concern, especially with objective physical activity measures. The possibility of missing data from individual studies included in this systematic review was outside the objectives of the present study, but is a salient point that should be considered for future investigations. It is also important to understand patient-level factors associated with missing data. The use of statistical techniques that address missing data, such as multiple imputation, is one approach to address missing physical activity data. Importantly, it has been shown that multiple imputation leads to precise estimates of predicting 30-day mortality risk in cardiac surgery patients when important clinical variables are missing, as compared to estimating risk with a complete case analysis.[32]

Limitations

One limitation to consider is that the patients included across the studies evaluated in this systematic review may have been different, as the recruitment criteria were not always clearly stated. A small sample of studies explicitly stated that they excluded those with physical limitations and healthcare providers may have advised higher risk patients to not participate in physical activity. There is also a limitation associated with the methodology of this systematic review: only studies written in English were included, raising the possibility that some studies were missed.

Conclusion

Due to the mixed findings in this systematic review, it cannot be concluded that self-reported physical activity behavior before cardiac surgery is associated with health outcomes after surgery. The mixed findings could be due to the heterogeneity in physical activity tools used, definitions of outcomes, and the

For peer review only

few studies adjusting for other potentially confounding variables. These findings highlight the need for more research in this area.

For peer review only

REFERENCES

1. Ferguson TB, Hammill BG, Peterson ED, DeLong ER, Grover FL, STS National Database Committee. A decade of change--risk profiles and outcomes for isolated coronary artery bypass grafting procedures, 1990-1999: a report from the STS National Database Committee and the Duke Clinical Research Institute. Society of Thoracic Surgeons. *Ann Thorac Surg*. 2002;73(2):480-489-490.
2. Pfisterer M, Buser P, Osswald S, et al. Outcome of elderly patients with chronic symptomatic coronary artery disease with an invasive vs optimized medical treatment strategy: one-year results of the randomized TIME trial. *JAMA*. 2003;289(9):1117-1123.
3. Alexander KP, Anstrom KJ, Muhlbaier LH, et al. Outcomes of cardiac surgery in patients > or = 80 years: results from the National Cardiovascular Network. *J Am Coll Cardiol*. 2000;35(3):731-738.
4. Anderson L, Oldridge N, Thompson DR, et al. Exercise-Based Cardiac Rehabilitation for Coronary Heart Disease: Cochrane Systematic Review and Meta-Analysis. *J Am Coll Cardiol*. 2016;67(1):1-12. doi:10.1016/j.jacc.2015.10.044.
5. Macchi C, Polcaro P, Cecchi F, et al. One-year adherence to exercise in elderly patients receiving postacute inpatient rehabilitation after cardiac surgery. *Am J Phys Med Rehabil Assoc Acad Physiatr*. 2009;88(9):727-734.
6. Horne D, Kehler DS, Kaoukis G, et al. Impact of Physical Activity on Depression After CardiacSurgery. *Can J Cardiol*. 2013;29(12):1649-1656.
7. Arthur HM, Daniels C, McKelvie R, Hirsh J, Rush B. Effect of a preoperative intervention on preoperative and postoperative outcomes in low-risk patients awaiting elective coronary artery bypass graft surgery. A randomized, controlled trial. *Ann Intern Med*. 2000;133(4):253-262.
8. Sawatzky JV, Kehler DS, Ready AE, et al. Prehabilitation program for elective coronary artery bypass graft surgery patients: a pilot randomized controlled study. *Clin Rehabil*. 2013;In press.
9. Herdy AH, Marcchi PL, Vila A, et al. Pre- and postoperative cardiopulmonary rehabilitation in hospitalized patients undergoing coronary artery bypass surgery: a randomized controlled trial. *Am J*

For peer review only

Phys Med Rehabil Assoc Acad Physiatr. 2008;87(9):714-719. doi:10.1097/PHM.0b013e3181839152.

10. Society of Thoracic Surgeons. General Thoracic Surgery Database. http://www.sts.org/sts-national-database/database-managers/general-thoracic-surgery-database. Accessed February 17, 2015.
11. Wells GA, Shea B, O'Connell D, et al. The Newcastle-Ottawa Scale (NOS) for assessing the quality of nonrandomised studies in meta-analyses. http://www.ohri.ca/programs/clinical_epidemiology/oxford.asp. Published 2011. Accessed January 25, 2015.
12. Markou AL, Lasten PJ, Noyez L. Physical activity post myocardial revascularization. Will surgery improve my mobility?. *J Cardiovasc Surg (Torino)*. 2007;48(2):201-206.
13. Nery RM, Barbisan JN, Mahmud MI. Influence of the practice physical activity in the coronary artery bypass graft surgery results. *Braz J Cardiovasc Surg*. 2007;22(3):297-302.
14. Markou AL, Evers M, van Swieten HA, Noyez L. Gender and physical activity one year after myocardial revascularization for stable angina. *Interact Cardiovasc Thorac Surg*. 2008;7(1):96-100.
15. Martini MR, Barbisan JN. Influence of physical activity during leisure time in patients in the follow-up two years after CABG. *Rev Bras Cir Cardiovasc Orgao Of Soc Bras Cir Cardiovasc*. 2010;25(3):359-364.
16. Nery RM, Barbisan JN. Effect of leisure-time physical activity on the prognosis of coronary artery bypass graft surgery. *Rev Bras Cir Cardiovasc Orgao Of Soc Bras Cir Cardiovasc*. 2010;25(1):73-78.
17. Rengo G, Galasso G, Vitale DF, et al. An active lifestyle prior to coronary surgery is associated with improved survival in elderly patients. *J Gerontol A Biol Sci Med Sci*. 2010;65(7):758-763. doi:10.1093/gerona/glp216.
18. Giaccardi M, MacChi C, Colella A, et al. Postacute rehabilitation after coronary surgery: The effect of preoperative physical activity on the incidence of paroxysmal atrial fibrillation. *Am J Phys Med Rehabil*. 2011;90(4):308-315.

For peer review only

19. Cacciatore F, Anello CB, Ferrara N, et al. Determinants of prolonged intensive care unit stay after cardiac surgery in the elderly. *Aging Clin Exp Res*. 2012;24(6):627-634.

20. Noyez L, Biemans I, Verkroost M, van Swieten H. Is a sedentary lifestyle an independent predictor for hospital and early mortality after elective cardiac surgery?. *Neth Heart J*. 2013;21(10):439-445.

21. Min L, Mazzurco L, Gure TR, et al. Longitudinal functional recovery after geriatric cardiac surgery. *J Surg Res*. 2015;194(1):25-33.

22. van Laar C, Kievit PC, Noyez L. Surgical aortic valve replacement in patients older than 75 years: is there really a quality of life benefit?. *Neth Heart J*. 2015;23(3):174-179.

23. van Poppel MN, Chinapaw MJ, Mokkink LB, van Mechelen W, Terwee CB. Physical activity questionnaires for adults: a systematic review of measurement properties. *Sports Med Auckl NZ*. 2010;40(7):565-600. doi:10.2165/11531930-000000000-00000;

24. Tremblay MS, Warburton DER, Janssen I, et al. New Canadian physical activity guidelines. *Appl Physiol Nutr Metab Physiol Appliquée Nutr Métabolisme*. 2011;36(1):36-46-58. doi:10.1139/H11-009.

25. Physical Activity Guidelines Advisory Committee. *Physical Activity Guidelines Advisory Committee Report.* Washington, DC: U.S.: Department of Health and Human Services; 2008.

26. Lee PH, Macfarlane DJ, Lam TH, Stewart SM. Validity of the International Physical Activity Questionnaire Short Form (IPAQ-SF): a systematic review. *Int J Behav Nutr Phys Act*. 2011;8. doi:10.1186/1479-5868-8-115.

27. Schmidt MD, Cleland VJ, Thomson RJ, Dwyer T, Venn AJ. A comparison of subjective and objective measures of physical activity and fitness in identifying associations with cardiometabolic risk factors. *Ann Epidemiol*. 2008;18(5):378-386. doi:10.1016/j.annepidem.2008.01.005.

28. Atienza AA, Moser RP, Perna F, et al. Self-reported and objectively measured activity related to biomarkers using NHANES. *Med Sci Sports Exerc*. 2011;43(5):815-821. doi:10.1249/MSS.0b013e3181fdfc32.

For peer review only

29. Anderson L, Thompson DR, Oldridge N, et al. Exercise-based cardiac rehabilitation for coronary heart disease. *Cochrane Database Syst Rev*. 2016;1:CD001800. doi:10.1002/14651858.CD001800.pub3.

30. Goldfarb M, Drudi L, Almohammadi M, et al. Outcome Reporting in Cardiac Surgery Trials: Systematic Review and Critical Appraisal. *J Am Heart Assoc*. 2015;4(8):e002204. doi:10.1161/JAHA.115.002204.

31. Abah U, Dunne M, Cook A, et al. Does quality of life improve in octogenarians following cardiac surgery? A systematic review. *BMJ Open*. 2015;5(4):e006904. doi:10.1136/bmjopen-2014-006904.

32. Karim MN, Reid CM, Tran L, Cochrane A, Billah B. Missing Value Imputation Improves Mortality Risk Prediction Following Cardiac Surgery: An Investigation of an Australian Patient Cohort. *Heart Lung Circ*. 2017;26(3):301-308. doi:10.1016/j.hlc.2016.06.1214.

For peer review only

FIGURE LEGENDS

Figure 1. Study flow diagram.

For peer review only

Table 1. Characteristics of included studies.

First author, year	Study Population	Country	Participants at follow-up	Physical Activity Assessment	Longest follow-up	Main findings
Giaccardi, 2011[18]	All patients ≥ 65 years undergoing CABG and/or valve procedures (total sample: 74.1 ± 5.8 years old); 43% female	Italy	158	Harvard Alumni Questionnaire	Four weeks postoperatively	Physical activity had an independent association with postoperative atrial fibrillation within 30 days.
Markou, 2007[12]	Elective CABG patients (Active: 64.4 ± 9.4, Inactive: 63.8 ± 9.0 years old); % female not reported	Netherlands	428	The Corpus Christi Heart Project	One year	Inactive vs. Active group had significantly more peri-operative MIs, but not reoperations, ICU LOS, HLOS, or postoperative complications at one year. Inactive group was more likely than Active group to be physically active at one year.
Nery, 2007[13]	All patients undergoing CABG (Active: 63 ± 11, Inactive 66 ± 14 years old); 42% female	Brazil	55	Structured Questionnaire confirmed by Minnesota Leisure Time Physical Activity Questionnaire	One year	Inactive vs. Active group had significantly longer HLOS and more postoperative events at one year.
Markou, 2008[14]	Elective CABG patients (64.3 ± 9.04 years old); 18% female	Netherlands	568	The Corpus Christi Heart Project	One year	Inactive vs. Active group were more likely to be more physically active one year postoperatively.
Martini, 2010[15]	Elective CABG patients (Active: 60 ± 10, Inactive: 62 ± 10 years old); 34% female	Brazil	185	Baecke Usual Physical Activity Questionnaire	Two years	Inactive vs. Active group did not have significantly different MACCE outcomes at two years.

For peer review only

Nery, 2010[16]	Elective CABG patients (Active: 60 ± 10, Inactive: 62 ± 10 years old); 34% female	Brazil	202	Baecke Usual Physical Activity Questionnaire	Hospital discharge	Inactive vs. Active group had more postoperative events within 30 days and a longer HLOS.
Rengo, 2010[17]	Acute or elective CABG patients ≥ 70 years (Active: 72.3 ± 3.2, Inactive: 76.1 ± 3.9 years old); 34% female	Italy	587	Physical Activity Scale for the Elderly	Mean 44.3 ± 21.0 months	Physical activity had an independent and dose association with cardiac and all-cause mortality five years postoperatively.
Cacciatore, 2012[19]	All patients ≥ 65 years undergoing CABG and/or valve procedures (72.9 ± 4.8 years old); 48% female	Italy	250	Physical Activity Scale for the Elderly	Hospital discharge	Physical activity was independently associated with reduced prolonged ICU LOS. Physical activity was not independently associated with postoperative ADLs.
Noyez, 2013[20]	Elective CABG and/or valve patients (69.7 ± 10.1 years old);	Netherlands	3150	The Corpus Christi Heart Project	30 days postoperatively	Physical activity was not independently associated with hospital or 30 day mortality. Inactive vs. Active group had a significantly longer ICU LOS.
Min, 2015[21]	Elective CABG and/or valve patients ≥ 65 years (74.7 ± 5.9 years old)	United States of America	62	The Health and Retirement Survey	4-6 months	Inactive vs. Active group had significantly higher postoperative physical activity up to 6 months postoperatively.
van Laar[22]	Patients ≥75 years undergoing elective isolated aortic valve replacement (79.5 ± 2.8 years old); 59% female	Netherlands	115	The Corpus Christi Heart Project	2 years postoperatively	Inactive vs. Active group had significantly higher mortality rates 2 years postoperatively.

CABG, coronary artery bypass graft surgery; HLOS, hospital length of stay; ICU LOS, intensive care unit length of stay; MI, myocardial infarction; MACCE, major adverse cerebrovascular and cardiac events; ADL, activities of daily living.

For peer review only

Table 2. Major adverse and cerebrovascular events and postoperative events within 30 days

Reference	Outcome definition	Adjustment variables	Number of events per group	OR or HR and 95% CI
**Major adverse cerebrovascular and cardiac events**				
Nery, 2007[13]	One year postoperative AF, hospital readmission, new CABG, PCI, MI	None	Active: 8/25 (31%); Inactive: 17/30 (57%)[a]	NR
Martini, 2010[15]	Two year postoperative death, re-hospitalization, cerebrovascular accident, MI	None	Active: 9/66 (14%); Inactive: 31/119 (26%)	NR
Rengo, 2010[17]	Five-year postoperative cardiac and all-cause mortality	Demographics, medical history, medications, and clinical findings.	NR	Adjusted proportional hazard models:<br>*All-cause mortality:* Exp(B) 0.248 (95% CI 0.141-0.434) [a]<br>*Cardiac mortality:*<br>Exp(B) 0.272 (0.133-0.555) [a]
van Laar 2015[22]	Two-year mortality	None	Active: 5/65 (13%); Inactive: 11/50 (22%)[a]	NR
**Postoperative events within 30 days**				
Markou, 2007[12]	Perioperative MI, Re-intervention, postoperative complications (wound, renal, neurological, pulmonary, gastrointestinal)	None	MI: Active: 4/226 (2%); Inactive: 11/202 (5%) [a]<br>Reoperation: Active: 15/226 (7%); Inactive: 9/202 (5%),<br>Wound infection: Active: 3/226 (1%); Inactive: 7/202 (3%),<br>Renal: Active: 3/226; Inactive: 7/202	NR
Nery, 2010[16]	Mortality, MI, reoperation	Age, smoking, PVD, COPD, Cleveland Risk Score.	Mortality: Active: 0/66 (0%); Inactive: 7/136 (5%)<br>MI: Active: 1/66 (2%); Inactive: 6/136 (4%)<br>Reoperation: Active: 0/66 (0%); Inactive: 1/136 (0.5%)	Multivariate OR for being active:<br>0.22 (95% CI 0.09-0.51, p=0.001)

For peer review only

Rengo, 2010[17]	Low-output syndromes, MI, cardiac support, stroke, bleedings, mediastinitis, pneumonia, dialysis	None	Any surgical complication: Active: 53/267 (19.7%); Inactive: 60/320 (18.6%)	NR
Giaccardi, 2011[18]	Atrial fibrillation	Age, episodes of AF one year preop, episodes of AF in the first week, β-blockers, amiodarone, left ventricular volume, left atrial emptying fraction	Postoperative atrial fibrillation: Active: 6/74 (8.1%); Inactive: 27/84 (32.1%)[a]	Multivariate OR for being inactive: 4.04 (95% CI 1.16-14.14, p=0.029)
Noyez, 2013[20]	Mortality, reoperation, stroke, renal insufficiency, sternal wound, ventilation	≥75 years, valve surgery, female, high operative risk, renal disease, obesity, NYHA IV, Insulin, vascular pathology, poor LVEF, lung disease, MI, neurological event	Hospital mortality: Active: 7/1815 (0.4%); Inactive: 15/1335 (1.1%)[a]<br>30 day mortality: Active: 10/1815 (0.6%); Inactive: 20/1335 (1.5%)[a]<br>Reoperation: Active: 105/1815 (5.8%); Inactive: 68/1335 (5%)<br>Stroke: Active: 9/1815 (0.5%); Inactive: 12/1335 (0.9%)<br>Renal insufficiency: Active: 32/1815 (1.8%); Inactive: 39/1335 (2.9%)[a]<br>Sternal wound: Active: 10/1815 (0.6%); Inactive: 17/1335 (1.3%)[a]<br>Ventilation >2 days: Active: 31/1815 (1.7%); Inactive: 54/1335 (4.0%)[a]	Hospital mortality multivariate OR for being inactive: 1.20 (95% CI 0.4-3.5, p=0.617)<br>30 day mortality multivariate OR for being inactive: 1.10 (95% CI 0.5-2.7, p=0.70)

[a] indicates statistical significance (P<0.05). CABG, coronary artery bypass graft; PCI, percutaneous coronary intervention; MI, myocardial infarction; NR, not reported; PVD, peripheral vascular disease; COPD, chronic obstructive pulmonary disease; OR, odds ratio; AF, atrial fibrillation; BMI, body mass index; NYHA, New York Heart Association; LVEF, left ventricular ejection fraction.

For peer review only

For peer review only

Table 3. Hospital length of stay, ICU length of stay, and postoperative activities of daily living and physical activity.

First author, year	Adjustment variables	Length of stay/number of events per group	Odds ratio (OR) or hazard ratio (HR) and 95% confidence interval (CI)
**Hospital length of stay**			
Markou, 2007[12]	None	Active: 6.9 ± 8.2 days; Inactive: 7.3 ± 7.1 days	NR
Nery, 2007[13]	None	Active: 12 ± 5 days, median 9 days (IQR 8-15); Inactive: 15 ± 8 days, median 12 (IQR 9-19)[a]	NR
Nery, 2010[16]	Age, sex, Cleveland Risk Score, smoking, systemic arterial hypertension, stroke, MI, and PVD.	NR	HR: 0.67 (95% CI 0.49-0.93)[a]
**ICU length of stay**			
Markou, 2007[12]	None	Active: 2.2 ± 5.3 days; Inactive: 2.1 ± 3.5 days	NR
Cacciatore, 2012[19]	For ICU LOS >3 days: age, off-pump CABG, stroke, renal failure.	Active: 2.58 ± 1.09 days; Inactive: 3.33 ± 1.68 days[a,b]	For ICU length of stay >3 days<br>Univariate OR: 0.984 (95% CI 0.977-0.992)[a]<br>Multivariate OR: 0.992 (95% CI 0.983-1.000)[a]
Noyez, 2013[20]	None	Active: 1.3 ± 1.9 days; Inactive 3.0 ± 41.8 days[a]<br><br>ICU > 5 days: Active: 19/1815 (1.0%); Inactive: 46/1335 (3.4%)[a]	NR
**Postoperative ADLs**			
Cacciatore,	Age, gender, CABG, NYHA ≥3,	NR	Beta: 0.099

2012[19]	ICU LOS ≥3 days, Off-pump CABG, diabetes, renal failure, stroke, PVD, COPD, Cumulative Illness Rating Scale.		
**Postoperative Physical activity**			
Markou, 2007[12]	Age ≥75 years, gender, neurological disease, vascular disease, diabetes, and preoperative physical activity.	Better PA post-operatively: Active: 48/226 (21.2 %), Inactive: 129/202 (64%) [a]<br><br>Equal PA post-operatively: Active: 112/226 (49.6%), Inactive: 59/202 (29.2%) [a]<br><br>Worse PA postoperatively: Active: 66/226 (29.2%), Inactive: 14/202 (6.9%) [a]	Decreased postoperative PA OR (inactive group as reference): 8.1 (95% CI 3.5-13.5) [a]
Markou, 2008[14]	Diabetes, vascular disease, neurological disease, renal disease, MI, preoperative activity level.	NR	For becoming physically inactive postoperatively<br>Male OR (inactive group as reference): 7.11 (95% CI 3.6-13.9) [a]<br><br>Female OR (inactive group as reference): 11.0 (95% CI 2.2-55) [a]
Min, 2015[21]	None	NR	Each weekly preoperative activity point was associated with a loss of 0.78 points at 6 weeks, p<0.001, and 0.65 points at 6 months) [a]

[a] indicates statistical significance (P<0.05). [b]Unpublished data obtained from Cacciatore et al, [19]. ICU, Intensive Care Unit; ADL; activities of daily living; IQR, interquartile range; NR, not reported; MI, myocardial infarction; PVD, peripheral vascular disease; HR, hazard ratio; OR, odds ratio; CABG, coronary artery bypass graft; NYHA, New York Heart Association; COPD, chronic obstructive pulmonary disease; PA, physical activity.

For peer review only - http://bmjopen.bmj.com/site/about/guidelines.xhtml

Table 4. Newcastle-Ottawa scale risk of bias scores

Reference	Selection	Comparability	Outcome	Total
Markou, 2007[12]	3	2	3	8
Nery, 2007[13]	3	0	2	5
Markou, 2008[14]	3	2	2	7
Martini, 2010[15]	3	0	2	5
Nery, 2010[16]	3	2	2	7
Rengo, 2010[17]	4	2	3	9
Giaccardi, 2011[18]	3	2	2	7
Cacciatore, 2012[19]	3	2	2	7
Noyez, 2013[20]	3	2	3	8
Min, 2015[21]	4	2	1	7
van Laar[22]	3	0	3	6
Average scores ± SD	3.18±0.40	1.45±0.93	2.27±0.65	6.91±1.22

Maximum scores are 4, 2, and 3 for selection, comparability, and outcome, respectively. Maximum total score is 9. A lower score within each category and for a total score indicates a higher risk of bias.

For peer review only

Box 1. Guidelines for physical activity measurement and outcome assessment in cardiac surgery patients: limitations and opportunities for future research

Drawbacks	Opportunity
**Physical activity**	
1. Heterogeneity in tools used across studies<br>2. Only subjective measures were used<br>3. Time of preoperative physical activity assessment was unclear in most studies	-use of objectively measured tools (e.g., pedometers, accelerometers) accompanied by a questionnaire which can produce data that can be compared across studies, such as step counts, intensity, and duration of physical activity.<br>-Capture physical activity behavior as soon as a patient is placed on a wait list, or in non-elective cases, as soon as possible prior to surgery.<br>-Physical activity should be assessed ideally over a 7 day period.<br>-Physical activity should be assessed by intensity and duration per week, and in steps per day.
**Outcomes**	
4. Heterogeneity in MACCE and postoperative events within 30 days definitions	-MACCE should be evaluated as a long-term outcome and defined as death, stroke, myocardial infarction, and the need for re-do cardiac surgery. Each outcome should be evaluated individually.<br>-30-day postoperative events should be evaluated using the STS checklist:[10] along with reasons, evaluate unexpected return to the operating room, complications due to pulmonary, cardiovascular, gastrointestinal, hematological, urologic, infection, neurological, and other important miscellaneous outcomes (e.g., unexpected admission to ICU, or other events requiring admission to operating room requiring anesthesia.<br>-re-hospitalization for any cause after cardiac surgery should also be added to outcomes.
5. No patient-oriented outcomes were assessed	-Capture postoperative health-related quality of life, mental health, pain, and cardiac symptoms using validated tools within the first 30 days and at least one-year postoperatively.
**Statistical procedures**	
6. Shortage of studies addressing confounders	-use multivariate analysis, including logistic or linear regression, or analysis of variance statistical procedures. Ensure that a power analysis is conducted prior to conducting the study.

MACCE, major adverse cerebrovascular and cardiac events. STS, Society of Thoracic Surgeons. ICU, intensive care unit.

For peer review only

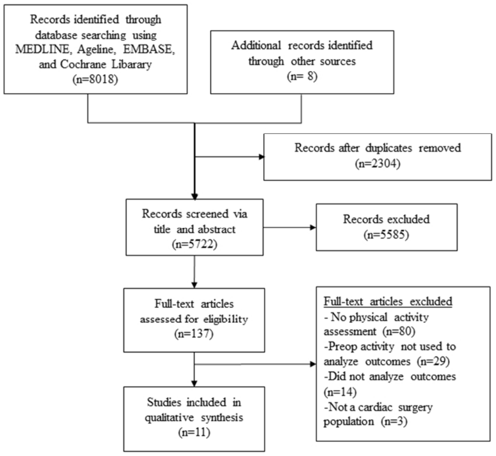

Figure 1. Study flow diagram

108x60mm (300 x 300 DPI)

review only

MEDLINE search strategy.

1. Sedentary Lifestyle/
2. physical endurance/ or physical fitness/
3. exercis*.mp. [mp=title, abstract, original title, name of substance word, subject heading word, keyword heading word, protocol supplementary concept word, rare disease supplementary concept word, unique identifier]
4. motor activit*.mp. [mp=title, abstract, original title, name of substance word, subject heading word, keyword heading word, protocol supplementary concept word, rare disease supplementary concept word, unique identifier]
5. accelerometry.mp. [mp=title, abstract, original title, name of substance word, subject heading word, keyword heading word, protocol supplementary concept word, rare disease supplementary concept word, unique identifier]
6. sedentary.mp. [mp=title, abstract, original title, name of substance word, subject heading word, keyword heading word, protocol supplementary concept word, rare disease supplementary concept word, unique identifier]
7. inactive.mp. [mp=title, abstract, original title, name of substance word, subject heading word, keyword heading word, protocol supplementary concept word, rare disease supplementary concept word, unique identifier]
8. (inactivity or physical inactivity or physical activity or active lifestyle or inactive lifestyle or physically active or physically inactive).mp. [mp=title, abstract, original title, name of substance word, subject heading word, keyword heading word, protocol supplementary concept word, rare disease supplementary concept word, unique identifier]
9. physical therapy modalities/ or exp exercise therapy/ or physiotherapy.mp. or physical therapy.mp. or motor activity/ or exp Exercise/ or physical exertion/ or physical endurance/ or anaerobic threshold/ or exercise tolerance/ or physical fitness/
10. (physical adj5 function*).mp. [mp=title, abstract, original title, name of substance word, subject heading word, keyword heading word, protocol supplementary concept word, rare disease supplementary concept word, unique identifier]
11. physical* mobil*.mp.
12. *"Quality of Life"/
13. 1 or 2 or 3 or 4 or 5 or 6 or 7 or 8 or 9 or 10 or 11 or 12
14. cardiac surger*.mp. [mp=title, abstract, original title, name of substance word, subject heading word, keyword heading word, protocol supplementary concept word, rare disease supplementary concept word, unique identifier]
15. cardiovascular surger*.mp. [mp=title, abstract, original title, name of substance word, subject heading word, keyword heading word, protocol supplementary concept word, rare disease supplementary concept word, unique identifier]
16. coronary artery bypass.mp. [mp=title, abstract, original title, name of substance word, subject heading word, keyword heading word, protocol supplementary concept word, rare disease supplementary concept word, unique identifier]
17. heart bypass.mp. [mp=title, abstract, original title, name of substance word, subject heading word, keyword heading word, protocol supplementary concept word, rare disease supplementary concept word, unique identifier]

For peer review only

18. ((aortic or aorta or mitral) adj7 (replacement or repair)).mp. [mp=title, abstract, original title, name of substance word, subject heading word, keyword heading word, protocol supplementary concept word, rare disease supplementary concept word, unique identifier]
19. cardiovascular surgical procedures/ or cardiac surgical procedures/ or cardiac valve annuloplasty/ or heart bypass, right/ or exp heart valve prosthesis implantation/ or myocardial revascularization/ or coronary artery bypass/ or coronary artery bypass, off-pump/ or vascular surgical procedures/
20. 14 or 15 or 16 or 17 or 18 or 19
21. (postoperative* or postoperative period or post surgical or post surgery).mp. or Postoperative Period/ or exp Postoperative Complications/
22. perioperative*.mp. or Perioperative Period/ or peroperative*.mp.
23. preoperative*.mp. or Preoperative Period/
24. Time Factors/
25. (after adj7 (surgery or bypass)).mp. [mp=title, abstract, original title, name of substance word, subject heading word, keyword heading word, protocol supplementary concept word, rare disease supplementary concept word, unique identifier]
26. (inpatient* or in hospital or hospitali* or discharge*).mp. [mp=title, abstract, original title, name of substance word, subject heading word, keyword heading word, protocol supplementary concept word, rare disease supplementary concept word, unique identifier]
27. 21 or 22 or 23 or 24 or 25 or 26
28. (pediatric* or paediatric* or child* or adolescen* or youth).mp. [mp=title, abstract, original title, name of substance word, subject heading word, keyword heading word, protocol supplementary concept word, rare disease supplementary concept word, unique identifier]
29. 13 and 20 and 27
30. 29 not 28

For peer review only

PRISMA 2009 Checklist

Section/topic	#	Checklist item	Reported on page #
**TITLE**			
Title	1	Identify the report as a systematic review, meta-analysis, or both.	1
**ABSTRACT**			
Structured summary	2	Provide a structured summary including, as applicable: background; objectives; data sources; study eligibility criteria, participants, and interventions; study appraisal and synthesis methods; results; limitations; conclusions and implications of key findings; systematic review registration number.	4
**INTRODUCTION**			
Rationale	3	Describe the rationale for the review in the context of what is already known.	6
Objectives	4	Provide an explicit statement of questions being addressed with reference to participants, interventions, comparisons, outcomes, and study design (PICOS).	6-7
**METHODS**			
Protocol and registration	5	Indicate if a review protocol exists, if and where it can be accessed (e.g., Web address), and, if available, provide registration information including registration number.	8
Eligibility criteria	6	Specify study characteristics (e.g., PICOS, length of follow-up) and report characteristics (e.g., years considered, language, publication status) used as criteria for eligibility, giving rationale.	8
Information sources	7	Describe all information sources (e.g., databases with dates of coverage, contact with study authors to identify additional studies) in the search and date last searched.	9
Search	8	Present full electronic search strategy for at least one database, including any limits used, such that it could be repeated.	9
Study selection	9	State the process for selecting studies (i.e., screening, eligibility, included in systematic review, and, if applicable, included in the meta-analysis).	8-9
Data collection process	10	Describe method of data extraction from reports (e.g., piloted forms, independently, in duplicate) and any processes for obtaining and confirming data from investigators.	10
Data items	11	List and define all variables for which data were sought (e.g., PICOS, funding sources) and any assumptions and simplifications made.	8
Risk of bias in individual studies	12	Describe methods used for assessing risk of bias of individual studies (including specification of whether this was done at the study or outcome level), and how this information is to be used in any data synthesis.	10
Summary measures	13	State the principal summary measures (e.g., risk ratio, difference in means).	No meta-analysis

For peer review only

PRISMA 2009 Checklist

Synthesis of results	14	Describe the methods of handling data and combining results of studies, if done, including measures of consistency (e.g., I^2) for each meta-analysis.	No meta-analysis

Section/topic	#	Checklist item	Reported on page #
Risk of bias across studies	15	Specify any assessment of risk of bias that may affect the cumulative evidence (e.g., publication bias, selective reporting within studies).	N/A
Additional analyses	16	Describe methods of additional analyses (e.g., sensitivity or subgroup analyses, meta-regression), if done, indicating which were pre-specified.	N/A
**RESULTS**			
Study selection	17	Give numbers of studies screened, assessed for eligibility, and included in the review, with reasons for exclusions at each stage, ideally with a flow diagram.	11
Study characteristics	18	For each study, present characteristics for which data were extracted (e.g., study size, PICOS, follow-up period) and provide the citations.	11 and in Table 1 (page 27-28)
Risk of bias within studies	19	Present data on risk of bias of each study and, if available, any outcome level assessment (see item 12).	15 and in Table 4 (page 33)
Results of individual studies	20	For all outcomes considered (benefits or harms), present, for each study: (a) simple summary data for each intervention group (b) effect estimates and confidence intervals, ideally with a forest plot.	8-10 and in tables 2 and 3 (pages 23-27)
Synthesis of results	21	Present results of each meta-analysis done, including confidence intervals and measures of consistency.	No meta-analysis
Risk of bias across studies	22	Present results of any assessment of risk of bias across studies (see Item 15).	N/A
Additional analysis	23	Give results of additional analyses, if done (e.g., sensitivity or subgroup analyses, meta-regression [see Item 16]).	N/A
**DISCUSSION**			
Summary of evidence	24	Summarize the main findings including the strength of evidence for each main outcome; consider their relevance to key groups (e.g., healthcare providers, users, and policy makers).	16-20

For peer review only - http://bmjopen.bmj.com/site/about/guidelines.xhtml

PRISMA 2009 Checklist

Limitations	25	Discuss limitations at study and outcome level (e.g., risk of bias), and at review-level (e.g., incomplete retrieval of identified research, reporting bias).	20
Conclusions	26	Provide a general interpretation of the results in the context of other evidence, and implications for future research.	20-21
**FUNDING**			
Funding	27	Describe sources of funding for the systematic review and other support (e.g., supply of data); role of funders for the systematic review.	2-3

From: Moher D, Liberati A, Tetzlaff J, Altman DG, The PRISMA Group (2009). Preferred Reporting Items for Systematic Reviews and Meta-Analyses: The PRISMA Statement. PLoS Med 6(6): e1000097. doi:10.1371/journal.pmed1000097

For more information, visit: www.prisma-statement.org.

For peer review only

For peer review only - http://bmjopen.bmj.com/site/about/guidelines.xhtml
