# Supplementary material for: Bangladesh Environmental Enteric Dysfunction (BEED) study: protocol for a community-based intervention study to validate non-invasive biomarkers of environmental enteric dysfunction
Source: BMJ Open. 2017 Aug 11;7(8):e017768. doi: 10.1136/bmjopen-2017-017768 (PMC5724211; doi:10.1136/bmjopen-2017-017768)
Supplement: Reviewer comments [file bmjopen-2017-017768.reviewer_comments.pdf]

PEER REVIEW HISTORY

BMJ Open publishes all reviews undertaken for accepted manuscripts. Reviewers are asked to complete a checklist review form (**http://bmjopen.bmj.com/site/about/resources/checklist.pdf**) and are provided with free text boxes to elaborate on their assessment. These free text comments are reproduced below.

ARTICLE DETAILS

**TITLE (PROVISIONAL)**	Bangladesh Environmental Enteric Dysfunction (BEED) study: Protocol for a community-based intervention study to validate non-invasive biomarkers of Environmental Enteric Dysfunction
**AUTHORS**	Mahfuz, Mustafa; Das, Subhasish; Mazumder, Ramendra; Rahman, M. Masudur; Haque, Rashidul; Bhuiyan, Md. Muzibur; Akhter, Hasina; Sarker, Md. Shafiqul; Mondal, Dinesh; Muaz, Syed Shafi; Karim, ASM Bazlul; Borowitz, Stephen M.; Moskaluk, Christopher A.; Barratt, Michael J.; Petri, William A.; Gordon, Jeffrey I.; Ahmed, Tahmeed

This article was not externally reviewed at BMJ Open. Protocols that have been independently assessed prior to submission to BMJ Open are usually fast-tracked to publication on the grounds that further substantial changes will not be possible. This independent assessment will usually be external, independent review for both a substantial grant award from a non-commercial or government funder and ethics approval.
